# Creation of High‐Performance Heterogeneous Photocatalysts by Controlling Ligand Desorption and Particle Size of Gold Nanocluster

**DOI:** 10.1002/anie.202104911

**Published:** 2021-07-01

**Authors:** Tokuhisa Kawawaki, Yuki Kataoka, Momoko Hirata, Yuki Akinaga, Ryo Takahata, Kosuke Wakamatsu, Yu Fujiki, Miori Kataoka, Soichi Kikkawa, Abdulrahman S. Alotabi, Sakiat Hossain, D. J. Osborn, Toshiharu Teranishi, Gunther G. Andersson, Gregory F. Metha, Seiji Yamazoe, Yuichi Negishi

**Affiliations:** ^1^ Department of Applied Chemistry Faculty of Science Tokyo University of Science Kagurazaka Shinjuku-ku Tokyo 162-8601 Japan; ^2^ Photocatalysis International Research Center Tokyo University of Science 2641 Yamazaki Noda Chiba 278-8510 Japan; ^3^ Institute for Chemical Research Kyoto University Gokasho Uji 611-0011 Japan; ^4^ Department of Chemistry Graduate School of Science Tokyo Metropolitan University 1-1 Minami-Osawa, Hachioji-shi Tokyo 192-0397 Japan; ^5^ Flinders Institute for Nanoscale Science and Technology Flinders University Adelaide South Australia 5042 Australia; ^6^ Department of Chemistry University of Adelaide Adelaide South Australia 5005 Australia

**Keywords:** catalysts, metal clusters, nanostructures, photocatalysts, water splitting

## Abstract

Recently, the creation of new heterogeneous catalysts using the unique electronic/geometric structures of small metal nanoclusters (NCs) has received considerable attention. However, to achieve this, it is extremely important to establish methods to remove the ligands from ligand‐protected metal NCs while preventing the aggregation of metal NCs. In this study, the ligand‐desorption process during calcination was followed for metal‐oxide‐supported 2‐phenylethanethiolate‐protected gold (Au) 25‐atom metal NCs using five experimental techniques. The results clearly demonstrate that the ligand‐desorption process consists of ligand dissociation on the surface of the metal NCs, adsorption of the generated compounds on the support and desorption of the compounds from the support, and the temperatures at which these processes occurred were elucidated. Based on the obtained knowledge, we established a method to form a metal‐oxide layer on the surface of Au NCs while preventing their aggregation, thereby succeeding in creating a water‐splitting photocatalyst with high activity and stability.

## Graphical abstract

The ligand‐desorption process during calcination was elucidated for metal‐oxide‐supported thiolate‐protected gold (Au) 25‐atom metal nanoculusters (NCs) using five experimental techniques. Furthermore, based on obtained knowledge, a method was established to form a metal‐oxide layer on the surface of Au NCs while preventing their aggregation, thereby succeeding in creating a water‐splitting photocatalyst with high activity and stability.

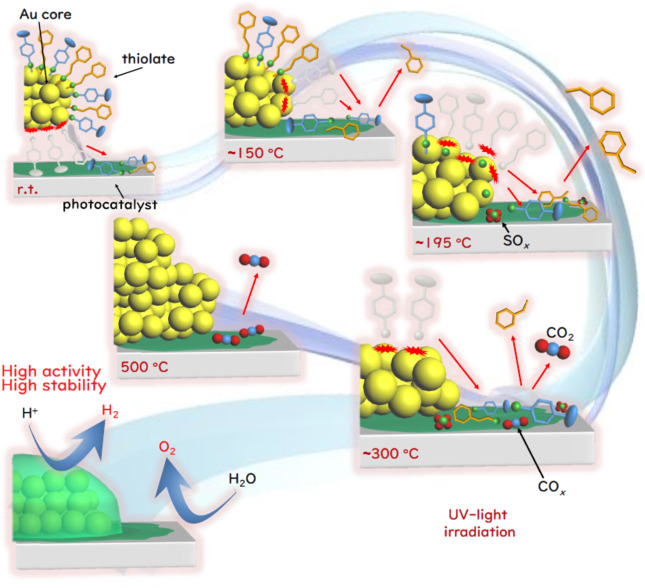

## Introduction

Recently, ligand‐protected metal nanoclusters controlled by atomic accuracy (atomically precise metal NCs)[^1^, ^2^, ^3^, ^4^, ^5^, ^6^, ^7^, ^8^, ^9^, ^10^, ^11^, ^12^, ^13^, ^14^] have been actively applied in heterogeneous catalysts (thermal‐, photo‐, and electro‐catalysts).[^15^, ^16^, ^17^, ^18^, ^19^, ^20^, ^21^, ^22^, ^23^, ^24^] Metal NCs exhibit physicochemical properties and functions that differ from those of the corresponding bulk metals and metal nanoparticles (NPs). Furthermore, their properties are dramatically affected by the number of constituent atoms and heteroatom substitutions.[^1^, ^2^, ^3^, ^4^, ^5^, ^6^, ^7^, ^8^, ^9^, ^10^, ^11^, ^12^, ^13^, ^14^] Therefore, novel heterogeneous catalysts with unique catalytic properties can be created using precise metal NCs. In addition, it is difficult to identify highly active particles in conventional heterogeneous catalysts because the metal NPs are loaded with a relatively large size distribution. In contrast, for heterogeneous catalysts loaded with atomically precise metal NCs, the chemical composition of the loaded metal NCs is defined, facilitating the identification of highly active metal NCs and their selective loading. For such heterogeneous catalysts, it is also easy to attain a deep understanding of the correlation between the geometric/interface structure, catalytic activity and the reaction mechanism.[^15^, ^16^, ^25^] Thus, the use of precise metal NCs in heterogeneous catalysis has many advantages for both the development of practical catalysts and the understanding of the mechanism of catalytic reactions.

**Scheme 1:**
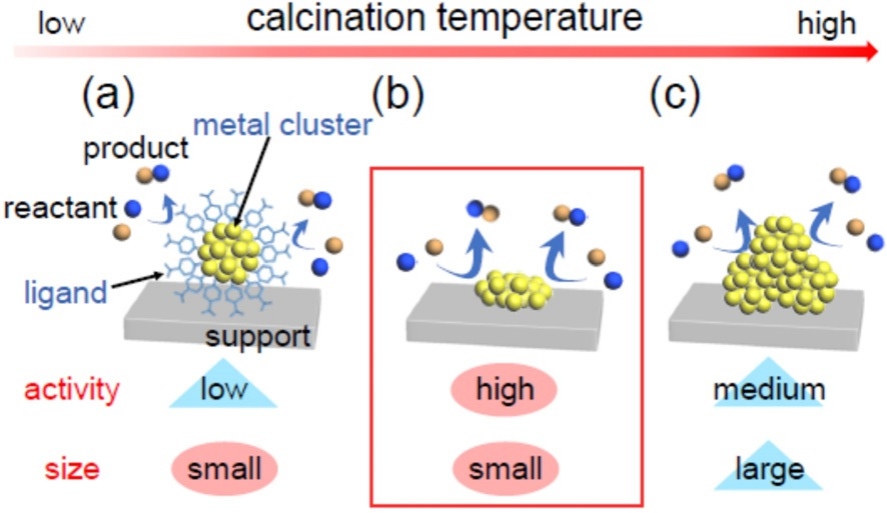
Schematic illustration of typical phenomena caused by increasing the calcination temperature in metal‐oxide‐supported ligand‐protected metal NCs: a) small size is maintained but low activity, b) high activity emerges while maintaining small size, and c) decreased activity due to aggregation.

When creating such heterogeneous catalysts, generally, 1) metal NCs are first precisely synthesized using ligands. Then, 2) the obtained atomically precise metal NCs are adsorbed onto the support. Typically, the presence of ligands in a heterogeneous catalyst leads to a decrease in catalytic activity because it inhibits the approach of the reactant to the surface of the metal NCs and induces a modification of the electronic structure of the metal NCs (Scheme 1(a)).[^26^, ^27^] Therefore, in many cases, 3) some or all of the ligands are removed by calcination or other pretreatments to attain higher activity (Scheme 1(b)).[^15^, ^16^, ^17^, ^18^, ^19^, ^20^, ^21^, ^22^, ^23^, ^24^, ^28^, ^29^] However, ligand removal also induces aggregation of metal NCs. When such aggregation occurs, the catalytic activity specific to metal NCs is diminished (Scheme 1(c)).[^15^, ^16^, ^17^, ^18^, ^19^, ^20^, ^21^, ^22^, ^23^, ^24^, ^28^, ^29^] Therefore, in ligand removal, it is extremely important to select conditions that remove only the ligands while maintaining the number of constituent atoms of the metal NCs. However, a clear understanding of the ligand‐desorption mechanism during calcination has not yet been attained. To perform calcination under appropriate conditions and therefore create a highly functional heterogeneous catalyst, it is essential to attain a deep understanding of this mechanism.

In this study, for metal‐oxide‐adsorbed 2‐phenylethanethiolate (PET; Scheme S1(a)) protected gold (Au) 25‐atom NCs ([Au_25_(PET)_18_]^−^; Scheme S2(a)), which is a commonly used catalyst in heterogeneous catalytic applications,[^18^, ^20^, ^21^, ^22^, ^23^, ^24^, ^25^, ^26^, ^27^, ^28^, ^29^, ^30^, ^31^, ^32^, ^33^] the ligand‐desorption process during calcination was followed using five experimental techniques. The results clearly demonstrate that the ligand‐desorption process consists of ligand dissociation on the surface of the metal NCs, adsorption of the generated compounds on the support and desorption of the compounds from the support, and elucidate the temperatures at which these processes occur. Based on the obtained knowledge, we have established a method to load Au NCs while preventing their aggregation, thereby succeeding in creating a water‐splitting photocatalyst with high activity and stability.

## Results and Discussion

### Ligand‐Desorption Mechanism

#### Flow of the Experiments

**Scheme 2:**
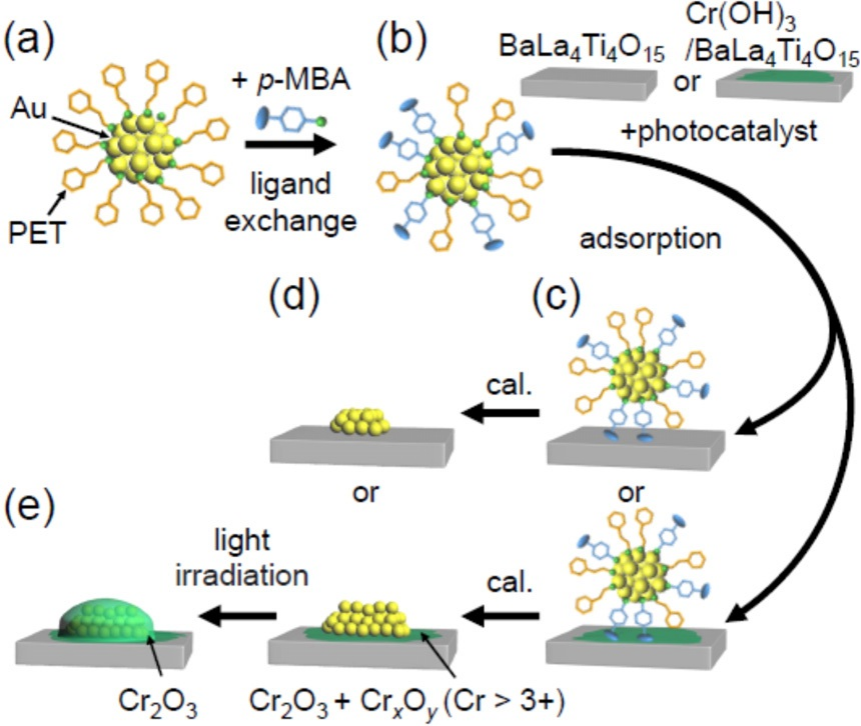
Schematic illustration of experimental procedure used in this work: a) synthesis of Au_25_(PET)_18_, b) preparation of Au_25_(PET, *p*‐MBA)_18_ using ligand‐exchange reaction, c) adsorption of Au_25_(PET, *p*‐MBA)_18_ on metal‐oxide support (Au_25_(PET, *p*‐MBA)_18_/BaLa_4_Ti_4_O_15_ or Au_25_(PET, *p*‐MBA)_18_/Cr(OH)_3_/BaLa_4_Ti_4_O_15_), d) ligand removal by calcination (Au_25_/BaLa_4_Ti_4_O_15_ or Au_25_/Cr(OH)_3_/BaLa_4_Ti_4_O_15_), and e) protection of Au NCs with Cr_2_O_3_ shell by light irradiation (Cr_2_O_3_/Au_25_/BaLa_4_Ti_4_O_15_).

The flow of the experiments is illustrated in Scheme 2. Further details for each experiment and measurement are provided in the Supporting Information.

**Scheme 3:**
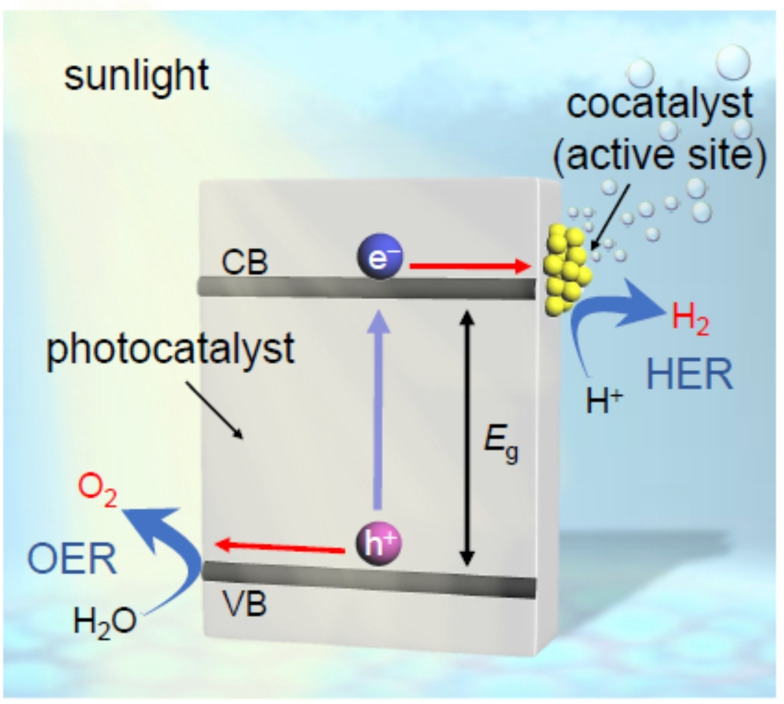
Schematic illustration of photocatalytic water splitting using a one‐step photoexcitation system. CB, conduction band; VB, valence band; *E*_g_, band gap. For the systems used in this work (i.e. Au_25_/BaLa_4_Ti_4_O_15_ and Cr_2_O_3_/Au_25_/BaLa_4_Ti_4_O_15_), the Au NCs only act as co‐catalysts and not as light absorbers.

For metal NCs, [Au_25_(PET)_18_]^−^ (counter ion is tetraoctylammonium ion=TOA^+^; hereinafter described as Au_25_(PET)_18_) was used. Au_25_(PET)_18_ was synthesized with atomic precision using a reported^[34]^ method with slight modification (Scheme 2(a), Scheme S3, and Figure S1A(a)). For metal oxides, to apply the obtained heterogeneous catalysts as water‐splitting photocatalysts (Scheme 3),[^35^, ^36^, ^37^, ^38^] BaLa_4_Ti_4_O_15_ (Scheme S2(b) and S4),[^39^, ^40^, ^41^, ^42^] which is one of the most advanced photocatalysts, was used. When metal oxides are placed in water, hydroxyl groups (‐OH) are generally formed on their surfaces. Metal NCs protected by hydrophobic ligands, such as PET, are barely adsorbed on such hydrophilic surfaces.^[43]^ However, to estimate the metal loading weight with high accuracy, it is necessary to adsorb the metal NCs on the support with a high adsorption efficiency. Therefore, some of the PET in Au_25_(PET)_18_ was replaced with hydrophilic *p*‐mercaptobenzoic acid (*p*‐MBA; Scheme S1(b))^[44]^ (Scheme 2(b) and Figure S1B(a)).^[43]^ The obtained Au_25_(PET)_18−*x*_(*p*‐MBA)_*x*_ (*x*=5–12; hereinafter described as Au_25_(PET, *p*‐MBA)_18_) was stirred with BaLa_4_Ti_4_O_15_ in acetone solution for 1 h at a weight ratio of 0.1 wt % Au, which gave the best water‐splitting photocatalytic activity in our previous study.^[40]^ Au_25_(PET, *p*‐MBA)_18_ was adsorbed on BaLa_4_Ti_4_O_15_ with an adsorption efficiency of more than 96 % (Au_25_(PET, *p*‐MBA)_18_/BaLa_4_Ti_4_O_15_; Scheme 2(c)). The same method was also used when Au_25_(PET, *p*‐MBA)_18_ was adsorbed on BaLa_4_Ti_4_O_15_, which was partially covered by an amorphous chromium hydroxide (Cr(OH)_3_) layer (Cr(OH)_3_/BaLa_4_Ti_4_O_15_) (Scheme 2(c)).

The ligands were removed from the catalyst by calcination (Scheme 2(d)). Specifically, Au_25_(PET, *p*‐MBA)_18_/BaLa_4_Ti_4_O_15_ or Au_25_(PET, *p*‐MBA)_18_/Cr(OH)_3_/BaLa_4_Ti_4_O_15_ was placed in an electric furnace and calcined under reduced pressure (Scheme S5). For the calcination temperature, it was increased from room temperature to each final temperature at a rate of ca. 7 °C min^−1^ and kept at the final temperature for 80 min. The sample obtained before and after the calcination was examined by direct insertion probe‐mass spectrometry (DIP‐MS; Scheme S6 and S7), X‐ray absorption fine structure (XAFS) analysis, Fourier‐transform infrared spectroscopy (FT‐IR), X‐ray photoelectron spectroscopy (XPS) analyses, and transmission electron microscopy (TEM).

### Mechanism for Au_25_(PET, p‐MBA)_18_

**Figure 1:**
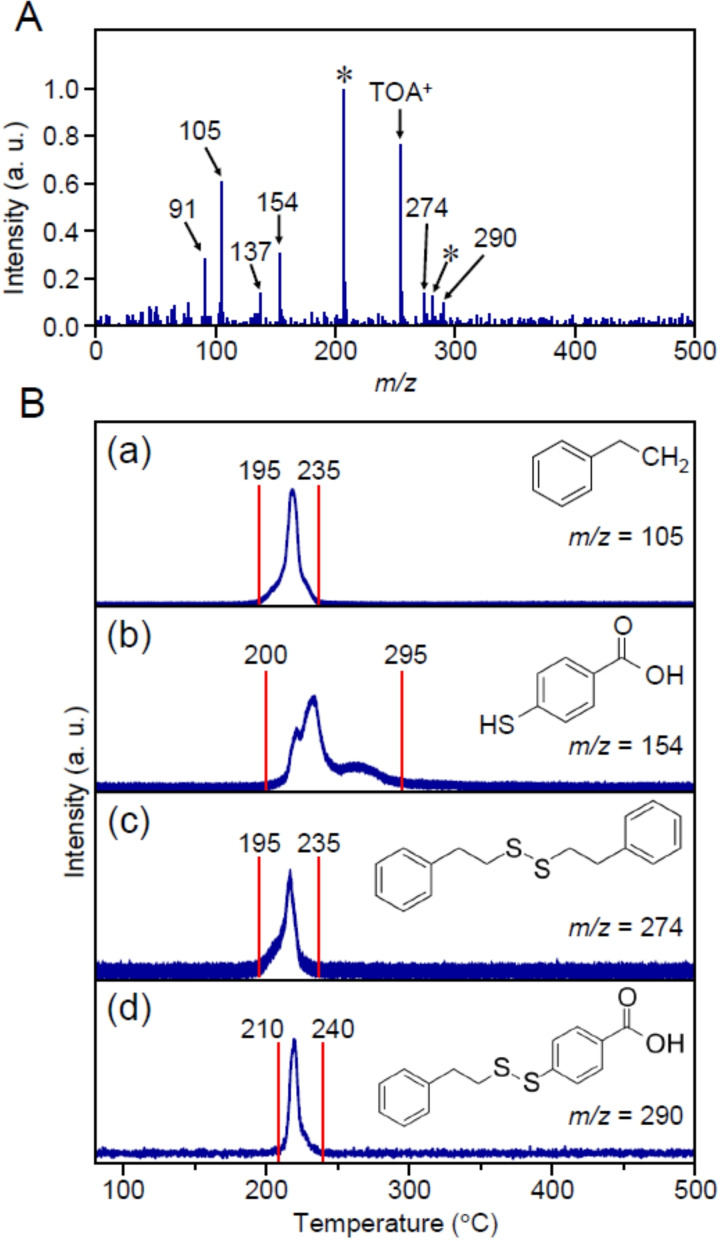
A) DIP‐MS spectrum of compounds desorbed from Au_25_(PET, *p*‐MBA)_18_ in the temperature range of 80–500 °C. In this spectrum, the peaks with an asterisk (*) are not due to the sample but to compounds deposited in the apparatus. B) Temperature dependence of each mass peak: a) PE (*m*/*z=*105), b) *p*‐MBA (*m*/*z=*154; not thiolate but thiol), c) (PET)_2_ (*m*/*z=*274), and d) PET−*p*‐MBA (*m*/*z=*290).

**Figure 2:**
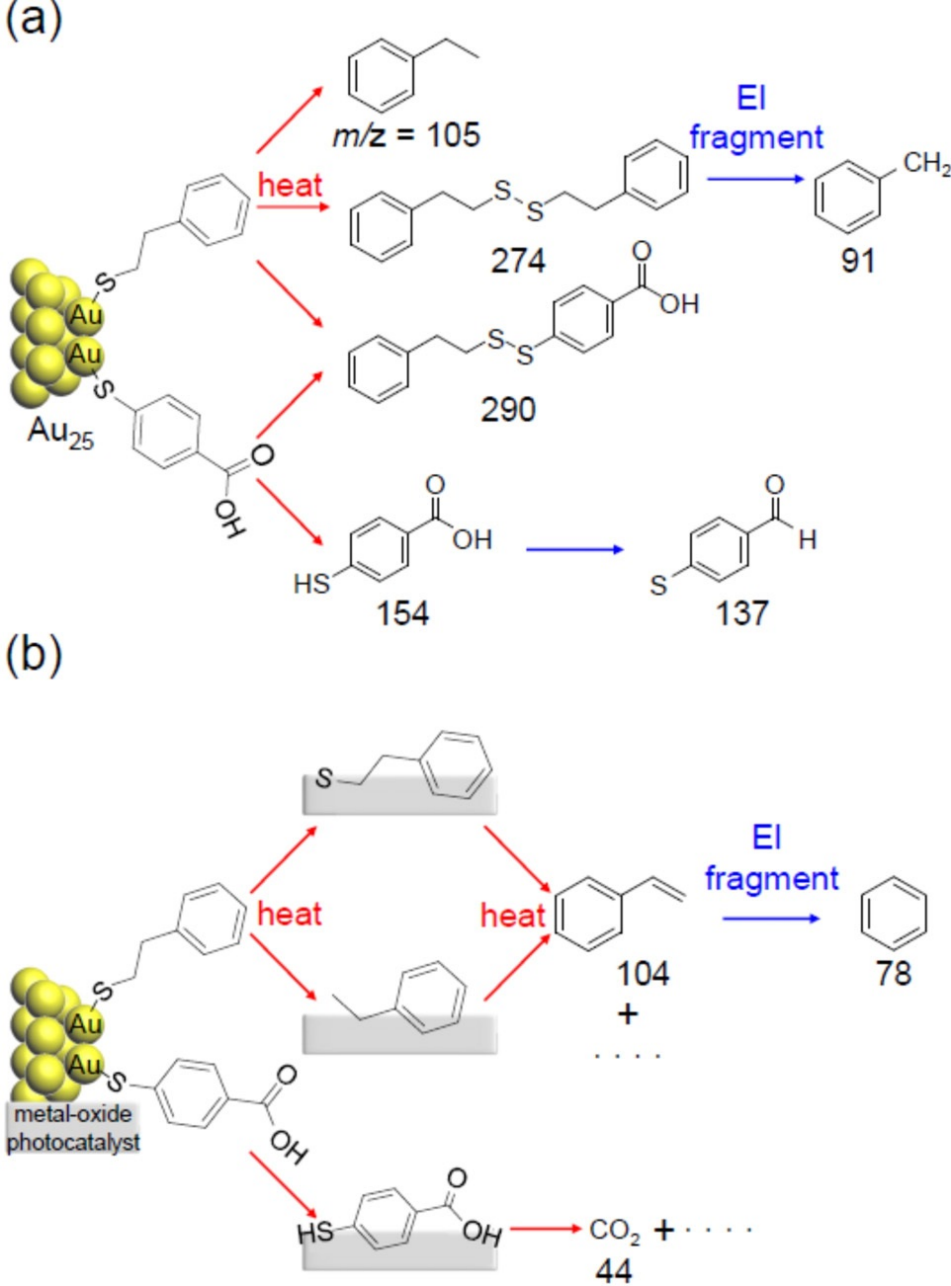
Origin of peaks observed in DIP‐MS spectra of a) Au_25_(PET, *p*‐MBA)_18_ and b) Au_25_(PET, *p*‐MBA)_18_/BaLa_4_Ti_4_O_15_ or Au_25_(PET, *p*‐MBA)_18_/Cr(OH)_3_/BaLa_4_Ti_4_O_15_. *p*‐MBA is desorbed from Au_25_ in the form of thiol and not in the form of thiolate.

To better understand the phenomena occurring on the metal oxide during calcination, we first examined the ligand‐desorption pattern of Au_25_(PET, *p*‐MBA)_18_, which was not loaded on the metal oxide. Figure 1 A presents the DIP‐MS spectrum of Au_25_(PET, *p*‐MBA)_18_. This MS spectrum contains peaks derived from all the compounds desorbed from the sample from 80 °C to 500 °C (Table S1). The main peaks appeared at *m*/*z*=91, 105, 137, 154, 254, 274, and 290. The peak at *m*/*z*=254 is attributed to a compound derived from TOA^+^ (Figure S2), which is the counter cation of Au_25_(PET, *p*‐MBA)_18_. Comparison of the DIP‐MS spectra with Au_25_(PET)_18_, Au_25_(PET, 3‐MPA)_18_, and Au_25_(SC4, *p*‐MBA)_18_ (3‐MPA=3‐mercaptopropionic acid; SC4=1‐buthanethiolate; Scheme S1(c)(d) and Figure S1) with different ligand combinations revealed that the peaks at *m*/*z*=91, 105, and 274 correspond to PET‐derived compounds, the peaks at *m*/*z*=137 and 154 correspond to *p*‐MBA‐derived compounds, and the peak at *m*/*z*=290 can be obtained only when both PET and *p*‐MBA are present (Figure S3–S5). It can be interpreted that the peak at *m*/*z*=91 is caused by EI dissociation of PET, and the peak at *m*/*z*=137 is caused by EI dissociation (Figure S6) of *p*‐MBA (M_W_=154) (Figure 2(a) and Figure S7). These results indicate that 1) calcination of Au_25_(PET, *p*‐MBA)_18_ yields phenylethane (PE; *m*/*z=*105), *p*‐MBA (*m*/*z*=154; in this case, thiol rather than thiolate), (PET)_2_ (*m*/*z*=274), and PET−*p*‐MBA (*m*/*z=*290) as the major desorbates, and 2) therefore, in the calcination of Au_25_(PET, *p*‐MBA)_18_, S−C and Au−S bond dissociations in Au−PET and Au−S bond dissociation in Au−*p*‐MBA occur as the main dissociation channels (Figure 2(a)). Although these results are overall consistent with previous reports,^[45]^ the fact that *p*‐MBA is desorbed from the surface of the Au NCs as a thiol rather than a thiolate was first demonstrated in this report.^[46]^

Figure 1 B(a)–(d) shows the correlation between the calcination temperature and ion intensity for PE (*m*/*z*=105), *p*‐MBA (*m*/*z*=154), (PET)_2_ (*m*/*z*=274), and PET−*p*‐MBA (*m*/*z*=290), respectively. PE was desorbed at 195–235 °C, *p*‐MBA was desorbed at 200–295 °C, (PET)_2_ was desorbed at 195–235 °C, and PET−*p*‐MBA was desorbed at 210–240 °C (Figure S8). These results indicate that in the calcination of Au_25_(PET, *p*‐MBA)_18_, S−C and Au−S dissociations of Au−PET begin to occur first, followed by Au−S dissociation of Au−*p*‐MBA. For *p*‐MBA, desorption was observed at several temperatures (Figure 1 B(b)). In Au_25_(PET, *p*‐MBA)_18_, there are two types of S sites (Scheme S2(a)). In addition, the temperature required for desorption is likely to differ depending on the state of the *p*‐MBAs, for example, the state in which the *p*‐MBAs are gathered or the state in which *p*‐MBA is located next to PET. The desorption of *p*‐MBA is considered to have occurred at multiple temperatures for these reasons.

### Mechanism for Au_25_(PET, p‐MBA)_18_/BaLa_4_Ti_4_O_15_

**Figure 3:**
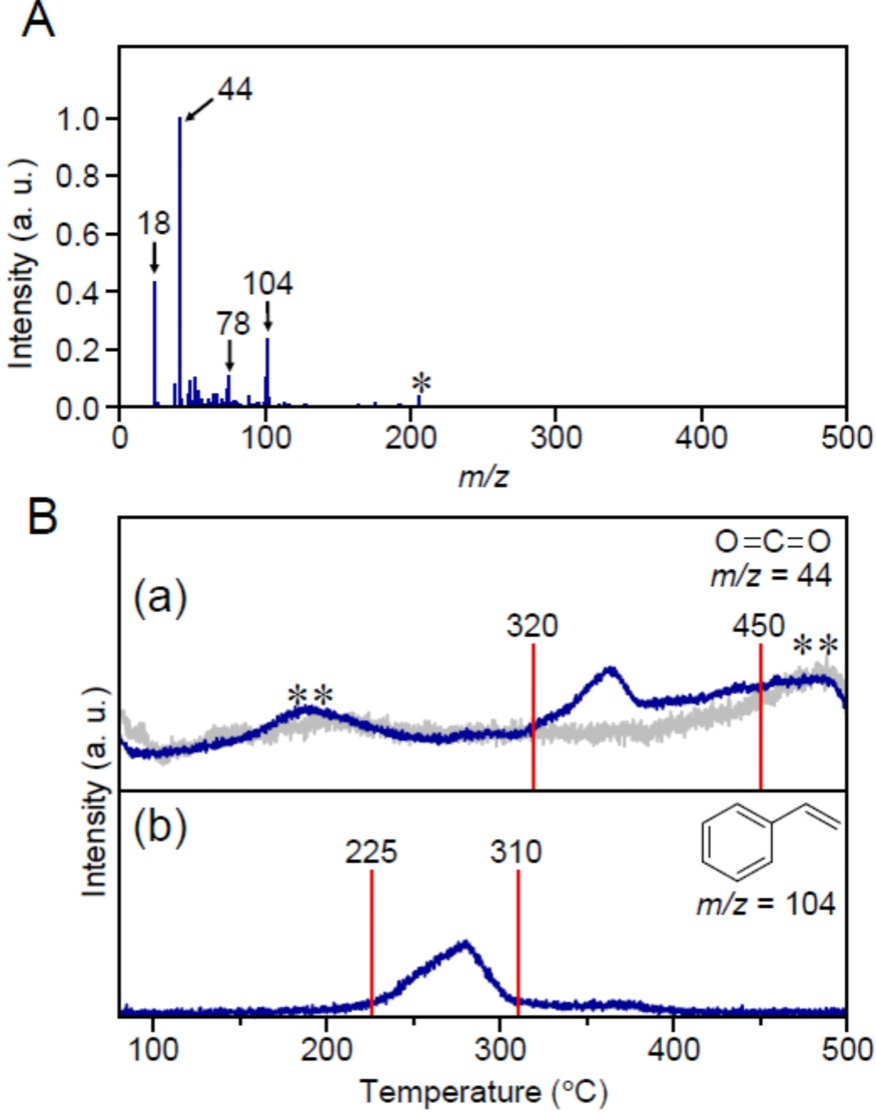
A) DIP‐MS spectrum of compounds desorbed from Au_25_(PET, *p*‐MBA)_18_/BaLa_4_Ti_4_O_15_ in the temperature range of 80–500 °C. In this spectrum, the peak at *m*/*z=*18 is assigned to water molecules adsorbed on BaLa_4_Ti_4_O_15_. The peak with the asterisk (*) is not due to the sample but to a compound deposited in the apparatus. B) Temperature dependence of each mass peak: a) CO_2_ (*m*/*z*=44) and b) styrene (*m*/*z*=104). In (B)(a), the peaks with double asterisk (**) were also observed in the calcination of only BaLa_4_Ti_4_O_15_ (gray line), implying that these peaks originated from organic compounds included in or attached to BaLa_4_Ti_4_O_15_.

Next, DIP‐MS measurements of Au_25_(PET, *p*‐MBA)_18_/BaLa_4_Ti_4_O_15_ were performed. Figure 3 A presents the mass spectra of the compounds desorbed at temperatures ranging from 80 °C to 500 °C. Surprisingly, the peaks attributed to PE (*m*/*z*=105), *p*‐MBA (*m*/*z*=154), (PET)_2_ (*m*/*z*=274), and PET−*p*‐MBA (*m*/*z*=290) were negligibly observed in the mass spectra. On the other hand, carbon dioxide (CO_2_; *m*/*z*=44), which is one of the final products of calcination, benzene (*m*/*z*=78), and styrene (*m*/*z*=104) were strongly observed in the mass spectra. Benzene is interpreted to form from the EI dissociation of styrene (Figure S9). Figure 3 B shows the correlation between the desorption temperature and ion intensity for CO_2_ (Figure 3 B(a)) and styrene (Figure 3 B(b)). The main desorption temperatures of CO_2_ (320–450 °C) and styrene (225–310 °C) were shifted to higher values compared with those for PE (195–235 °C), *p*‐MBA (200–295 °C), (PET)_2_ (195–235 °C), and PET−*p*‐MBA (210–240 °C) desorbed from unsupported Au_25_(PET, *p*‐MBA)_18_ (Figure 1 B and S10). These results imply that the PE, *p*‐MBA, (PET)_2_, and PET−*p*‐MBA thermally dissociated from Au_25_(PET, *p*‐MBA)_18_ were once adsorbed on the BaLa_4_Ti_4_O_15_ surface and then desorbed from the surface of BaLa_4_Ti_4_O_15_ in the form of styrene or CO_2_ (Figure 2(b)).

**Figure 4:**
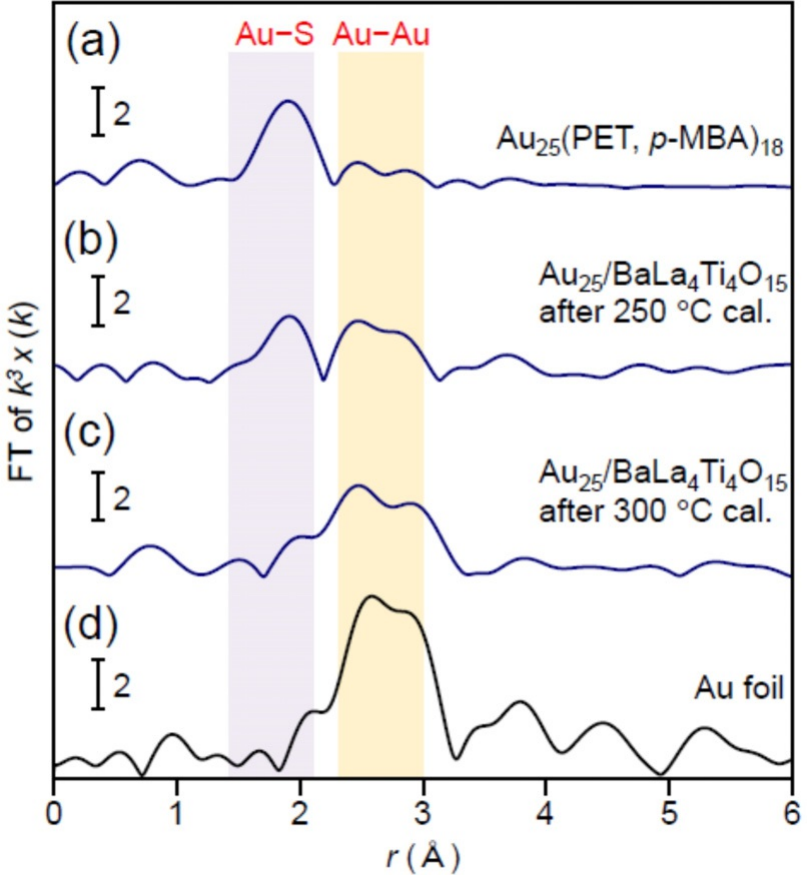
Au L_3_‐edge FT‐EXAFS spectra of a) Au_25_(PET, *p*‐MBA)_18_, Au_25_/BaLa_4_Ti_4_O_15_ obtained by calcination at b) 250 °C and c) 300 °C, and d) Au foil. The purple and yellow regions indicate the Au−S and Au−Au bond regions, respectively.[^43^, ^47^] In (a), only weak peaks appear in the Au−Au bond region because the Au_13_ core (Scheme S2(a)) fluctuates at room temperature.^[48]^

Au L_3_‐edge FT‐EXAFS analysis was performed on unsupported Au_25_(PET, *p*‐MBA)_18_ and calcined samples to attain a deeper understanding of the temperature at which each step occurs (Figure 4 and S11). The peaks at ca. 1.8 Å[^43^, ^47^, ^48^] attributed to the Au−S bond were clearly observed in the spectra of Au_25_(PET, *p*‐MBA)_18_ (Figure 4(a)) and the sample after calcination at 250 °C (Figure 4(b)). On the other hand, for the sample calcined at 300 °C (Figure 4(c)), the intensity of this peak was significantly reduced. This finding indicates that almost all the Au−S bonds dissociate in the temperature range of 250–300 °C in Au_25_(PET, *p*‐MBA)_18_/BaLa_4_Ti_4_O_15_, which is consistent with the results in Figure 1 B(b)–(d).

**Figure 5:**
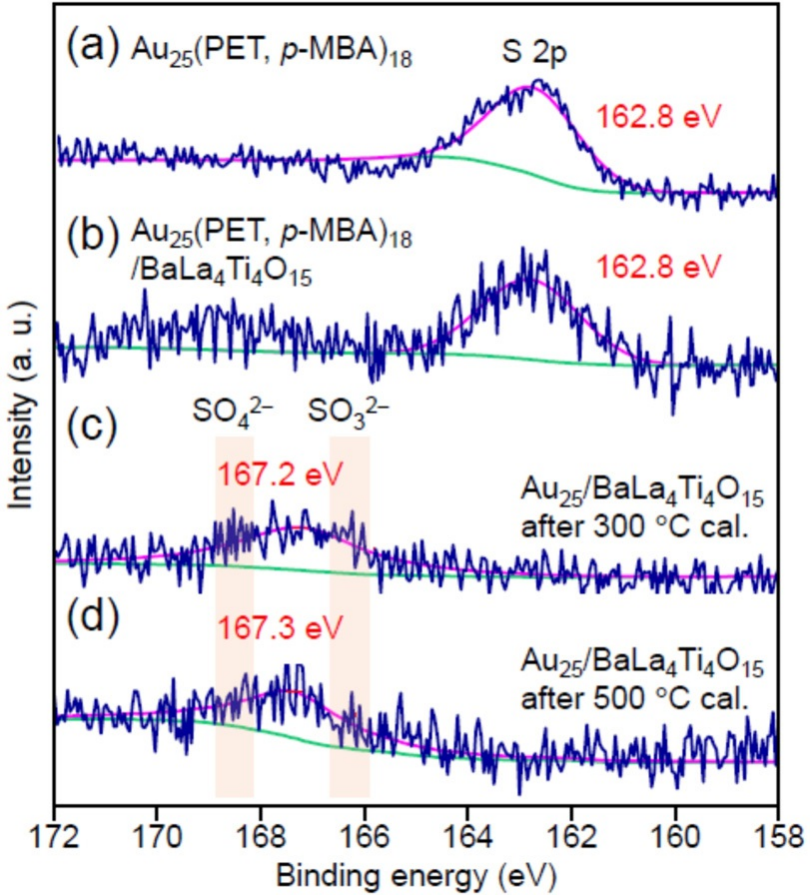
Comparison of S 2p XPS spectra: a) Au_25_(PET, *p*‐MBA)_18_, b) Au_25_(PET, *p*‐MBA)_18_/BaLa_4_Ti_4_O_15_, Au_25_/BaLa_4_Ti_4_O_15_ obtained by calcination at c) 300 °C and d) 500 °C. In the spectra, the green and purple lines indicate the baseline and fitting result, respectively. The peak at ca. 162.8 eV is assigned to S in Au−S, whereas the peaks at ca. 167.2 eV and ca. 167.3 eV are assigned to S oxides, such as SO_3_^2−^ and SO_4_^2−^.

As for the behavior of the compounds transferred onto BaLa_4_Ti_4_O_15_, it can be judged from Figure 3 B that the organic compounds start to be removed from the BaLa_4_Ti_4_O_15_ surface at 225 °C. Since the desorption of the compound from Au_25_(PET, *p*‐MBA)_18_ continues up to a temperature of ca. 300 °C (Figure 1 B and Figure 4), it can be interpreted that the migration of the compounds from Au_25_(PET, *p*‐MBA)_18_ onto BaLa_4_Ti_4_O_15_ and the desorption of organic compounds from the BaLa_4_Ti_4_O_15_ surface proceed in parallel at temperatures above 225 °C. With respect to S compounds, it was observed that the S compounds remain on the surface of BaLa_4_Ti_4_O_15_ in the form^[49]^ of SO_3_^2−^ or SO_4_^2−^ even at 500 °C (Figure 5).

**Figure 6:**
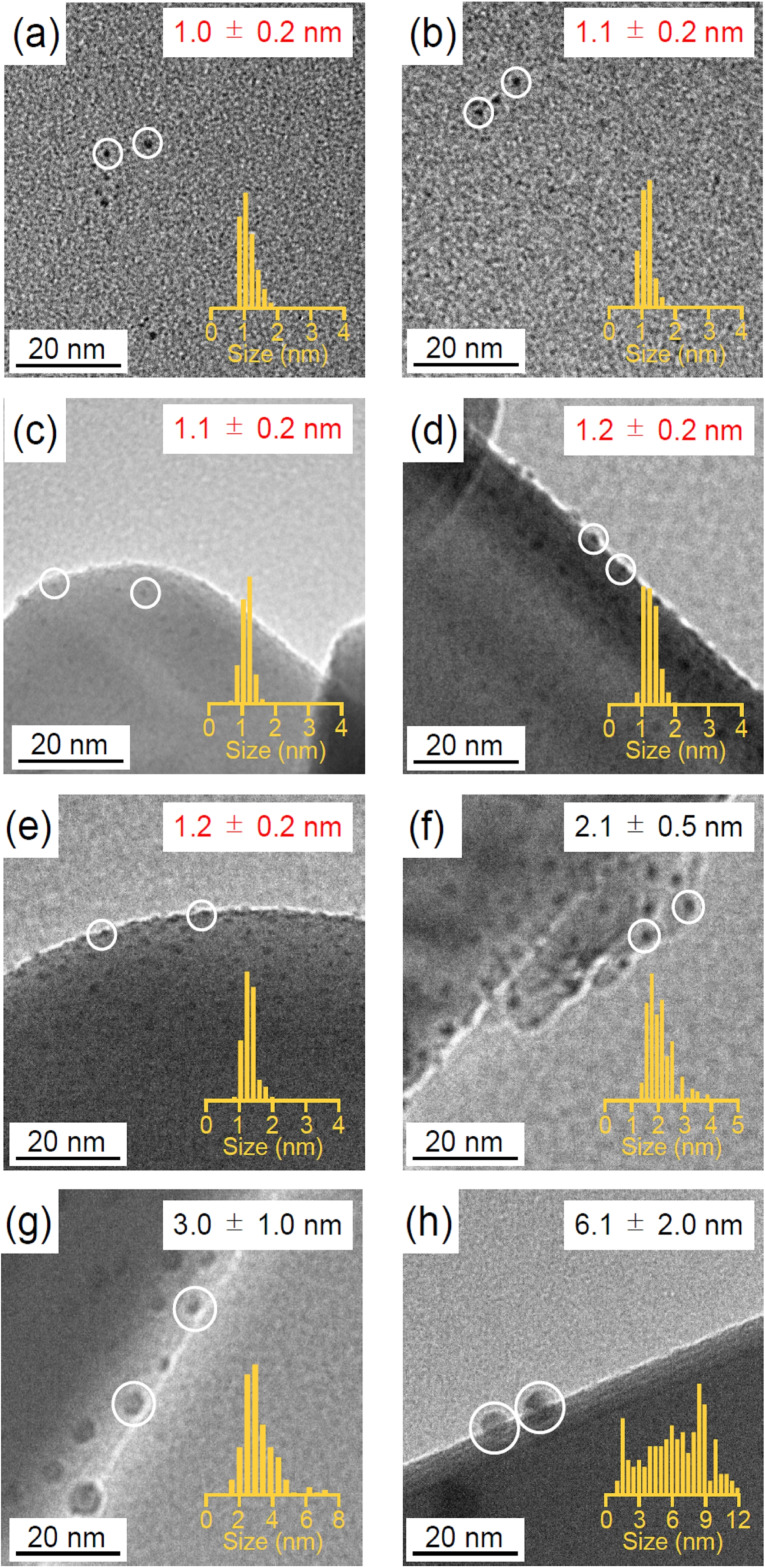
Comparison of TEM images and histograms; a) Au_25_(PET)_18_, b) Au_25_(PET, *p*‐MBA)_18_, c) Au_25_(PET, *p*‐MBA)_18_/BaLa_4_Ti_4_O_15_, Au_25_/BaLa_4_Ti_4_O_15_ obtained by calcination at d) 250 °C, e) 300 °C, f) 350 °C, g) 400 °C, and h) 500 °C.

Figure 6(a)—(h) present TEM images of Au_25_(PET)_18_, Au_25_(PET, *p*‐MBA)_18_, the sample before calcination, the sample after calcination at 250 °C, 300 °C, 350 °C, 400 °C, and 500 °C, respectively. In Figure 6(a)—(e), only fine particles of approximately 1 nm are observed. On the other hand, in Figure 6(f)—(h), particles with a size over 2 nm are observed. These results indicate that calcination up to 300 °C causes almost no aggregation of Au_25_; however, calcination at a higher temperature causes Au_25_ aggregation.

**Figure 7:**
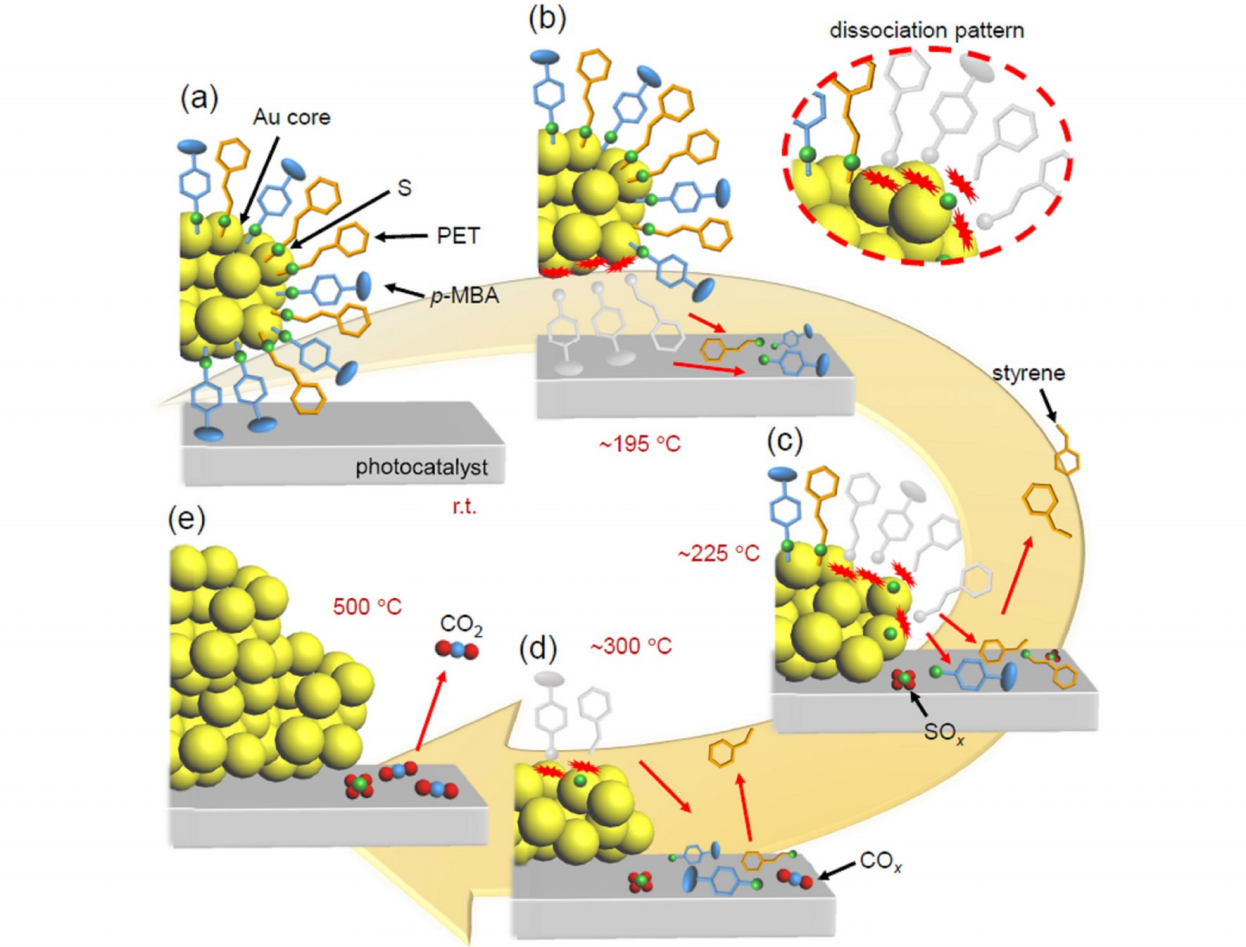
Proposed phenomenon occurring at each temperature during calcination of Au_25_(PET, *p*‐MBA)_18_/BaLa_4_Ti_4_O_15_; at a) room temperature, b) ca. 195 °C, c) ca. 225 °C, d) ca. 300 °C, and e) 500 °C.

Based on all the above results, the phenomena that occurs during calcination of Au_25_(PET, *p*‐MBA)_18_/BaLa_4_Ti_4_O_15_ can be described as follows (Figure 7):

1. The S−C and Au−S bond dissociation of Au−PET starts to occur at approximately 195 °C, followed by the Au−S dissociation of Au−*p*‐MBA at approximately 200 °C. These dissociations are complete by 295 °C. The compounds produced by the dissociation (PE, *p*‐MBA, (PET)_2_, and PET−*p*‐MBA) migrate to the BaLa_4_Ti_4_O_15_ surface (Figure 7(b)).
2. At temperatures above 225 °C, in parallel with 1), the adsorbates on BaLa_4_Ti_4_O_15_ start to desorb from the surface of BaLa_4_Ti_4_O_15_ in the form of styrene and/or CO_2_ (Figure 7(c)).
3. At ca. 300 °C, most of the ligands are desorbed from the surface of the Au NCs; the Au_25_ maintains its size even at this temperature (Figure 7(d)). However, based on our previous work, the geometric and electronic structures of Au_25_ is considered to change, largely due to elimination of the ligands; for example, the gold core geometry changes from a spherical structure (Scheme S2(a)) to a flat structure.^[43]^
4. A further increase of the calcination temperature causes significant aggregation of Au_25_ (Figure 7(e)). Some organic (Figure 3 B(a)) and S compounds (Figure 5(d)) continue to remain on BaLa_4_Ti_4_O_15_ and cannot be completely eliminated even at 500 °C (Figure 7(e)).

In addition to the strength of the Au−S and S−C bonds, the interaction between the ligands on the surface of the Au NCs also significantly affects the temperature of the ligand desorption from the surface of the Au NCs (Figure S12). It is also presumed that the temperature at which the compound is desorbed from the support is related to the magnitude of the compound‐support interaction.^[20]^ In addition, the ease of dissociation/desorption of the ligands and the resulting aggregation of Au NCs appears to slightly vary depending on the calcination atmosphere (Figure S13).[^20^, ^21^] However, the results suggesting 1)–4) have often been observed during previous calcinations performed with thiolate (SR) functional groups, supports, and atmospheres different from this study:[^20^, ^30^, ^40^, ^49^, ^50^, ^51^, ^52^] for example, Au_25_(SG)_18_/BaLa_4_Ti_4_O_15_ (SG=glutathionate) and Au_38_(PET)_24_/CeO_2_ (CeO_2_=cerium(IV) oxide). Therefore, although there are differences in the required temperatures, it is inferred that behavior similar to that described in 1)–4) occurs during the calcination of any SR‐protected Au NCs (Au_*n*_(SR)_*m*_ NCs; *n*=number of Au, *m*=number of SR ligands)/metal oxide. To date, a unified view has not been presented for the behavior of these Au_*n*_(SR)_*m*_ NCs/metal oxides during calcination.[^49^, ^53^] In this study, we succeeded in elucidating the details of the phenomena occurring during the calcination of Au_25_(PET, *p*‐MBA)_18_/BaLa_4_Ti_4_O_15_ by combining multiple experimental techniques (DIP‐MS, EXAFS spectroscopy, XPS, and TEM observation).

### Toward the Creation of High‐Performance Water‐Splitting Photocatalysts

As described above, the behavior of Au_25_(PET, *p*‐MBA)_18_/BaLa_4_Ti_4_O_15_ during calcination was elucidated, and most of the ligands were successfully removed from Au_25_ with almost no aggregation (Figure 6(e)). However, Au NCs with exposed surfaces are prone to aggregation when left untended (Figure S14) and during catalytic reactions.^[40]^ Therefore, to create a highly durable heterogeneous catalyst, it is essential to apply some type of treatment to the catalyst to suppress the aggregation of Au NCs. In our previous study, we showed that when Au_25_/Cr_2_O_3_/BaLa_4_Ti_4_O_15_, which was obtained by the calcination of Au_25_(PET, *p*‐MBA)_18_/Cr(OH)_3_/BaLa_4_Ti_4_O_15_, was irradiated with UV light, Au_25_ was embedded in the Cr_2_O_3_ layer (Cr_2_O_3_/Au_25_/BaLa_4_Ti_4_O_15_; Scheme 2(e)), and the stability of Au_25_ against aggregation was greatly improved.^[43]^ Furthermore, the formation of such a Cr_2_O_3_ film^[54]^ suppressed the reverse reaction on the surface of the Au NCs, resulting in higher water‐splitting activity.^[43]^ In the precursor, Au_25_(PET, *p*‐MBA)_18_, the ligand was strongly bound to the surface of the Au NCs. However, in Cr_2_O_3_/Au_25_/BaLa_4_Ti_4_O_15_, it is assumed that the amorphous structure of Cr_2_O_3_ is weakly bound to Au NCs and forms an overlying structure on Au NCs because Au does not form bonds with O easily.[^43^, ^55^] This appears to be the reason why Cr_2_O_3_/Au_25_/BaLa_4_Ti_4_O_15_ showed high water‐splitting activity without losing the high H_2_‐generation activity of small Au NCs. It has been reported by other groups that the formation of such metal/semiconductor oxide films on the surface of metal NCs improves the stability of metal NCs against not only photocatalytic water‐splitting reactions but also thermocatalytic reactions.[^17^, ^56^, ^57^] Therefore, the establishment of a method to form a metal oxide film on the Au_25_ surface while suppressing the aggregation of Au_25_ is expected to be extremely useful not only for the creation of high‐performance water‐splitting photocatalysts but also for the creation of high‐performance heterogeneous catalysts. In our previous study, the aggregation of Au_25_ occurred in the Cr_2_O_3_ layer.^[43]^ In the current study, we attempted to elucidate the behavior of Au_25_(PET, *p*‐MBA)_18_/Cr(OH)_3_/BaLa_4_Ti_4_O_15_ during calcination and light irradiation using six experimental techniques (DIP‐MS, EXAFS spectroscopy, FT‐IR spectroscopy, XPS, TEM observation, and HAADF‐STEM EDX element mapping), and then, based on the obtained knowledge, we sought to establish a method to better control the particle size of Au NCs.

**Figure 8:**
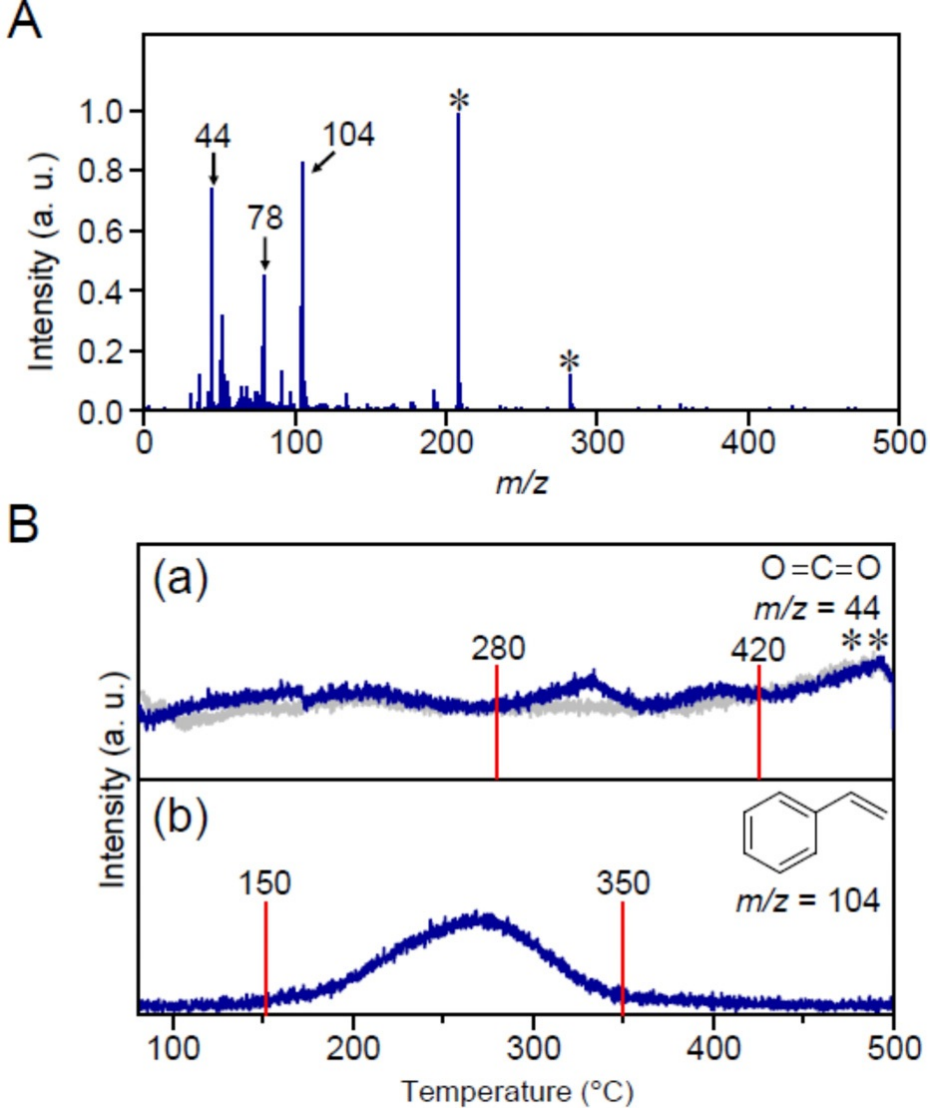
(A) DIP‐MS spectrum of the compounds desorbed from Au_25_(PET, *p*‐MBA)_18_/Cr(OH)_3_/BaLa_4_Ti_4_O_15_ in the temperature range of 80–500 °C. In this spectrum, the peaks with an asterisk (*) are not due to the sample but to compounds deposited in the apparatus. (B) Temperature dependence of each mass peak: (a) CO_2_ (*m*/*z=*44) and (b) styrene (*m*/*z=*104). In (B)(a), the peaks with a double asterisk (**) were also observed in the calcination of only BaLa_4_Ti_4_O_15_ (gray line), implying that these peaks originated from organic compounds included in or attached on BaLa_4_Ti_4_O_15_.

First, we investigated the behavior of Au_25_(PET, *p*‐MBA)_18_/Cr(OH)_3_/BaLa_4_Ti_4_O_15_ during calcination. Figure 8 A presents the DIP‐MS spectrum of Au_25_(PET, *p*‐MBA)_18_/Cr(OH)_3_/BaLa_4_Ti_4_O_15_ obtained by increasing the temperature from 80 °C to 500 °C. The observed compounds were overall very similar to those observed for Au_25_(PET, *p*‐MBA)_18_/BaLa_4_Ti_4_O_15_ (Figure 3 A). This result indicates that the overall mechanism of ligand removal during the calcination of Au_25_(PET, *p*‐MBA)_18_/Cr(OH)_3_/BaLa_4_Ti_4_O_15_ is similar to that of Au_25_(PET, *p*‐MBA)_18_/BaLa_4_Ti_4_O_15_ described in Figure 7. Indeed, the Au L_3_‐edge FT‐EXAFS (Figure S15) and S 2p XPS (Figure S16) results strongly support this interpretation.

However, there are also some differences in the calcination mechanism between Au_25_(PET, *p*‐MBA)_18_/BaLa_4_Ti_4_O_15_ and Au_25_(PET, *p*‐MBA)_18_/Cr(OH)_3_/BaLa_4_Ti_4_O_15_. For example, during the calcination of Au_25_(PET, *p*‐MBA)_18_/Cr(OH)_3_/BaLa_4_Ti_4_O_15_, styrene desorption started at a lower temperature (150 °C; Figure 8 B(b)) than that at which bond dissociation started on the surface of the Au NCs (195 °C; Figure 1 B). The study using FT‐IR spectroscopy (Figure S17 and S18) revealed that some of the ligands in Au_25_(PET, *p*‐MBA)_18_/Cr(OH)_3_/BaLa_4_Ti_4_O_15_ migrated from Au_25_(PET, *p*‐MBA)_18_ to Cr(OH)_3_/BaLa_4_Ti_4_O_15_ without heating. Such ligand migration is interpreted to be related to the start of the styrene desorption at 150 °C in Au_25_(PET, *p*‐MBA)_18_/Cr(OH)_3_/BaLa_4_Ti_4_O_15_ (Figure S19 and S20).

**Figure 9:**
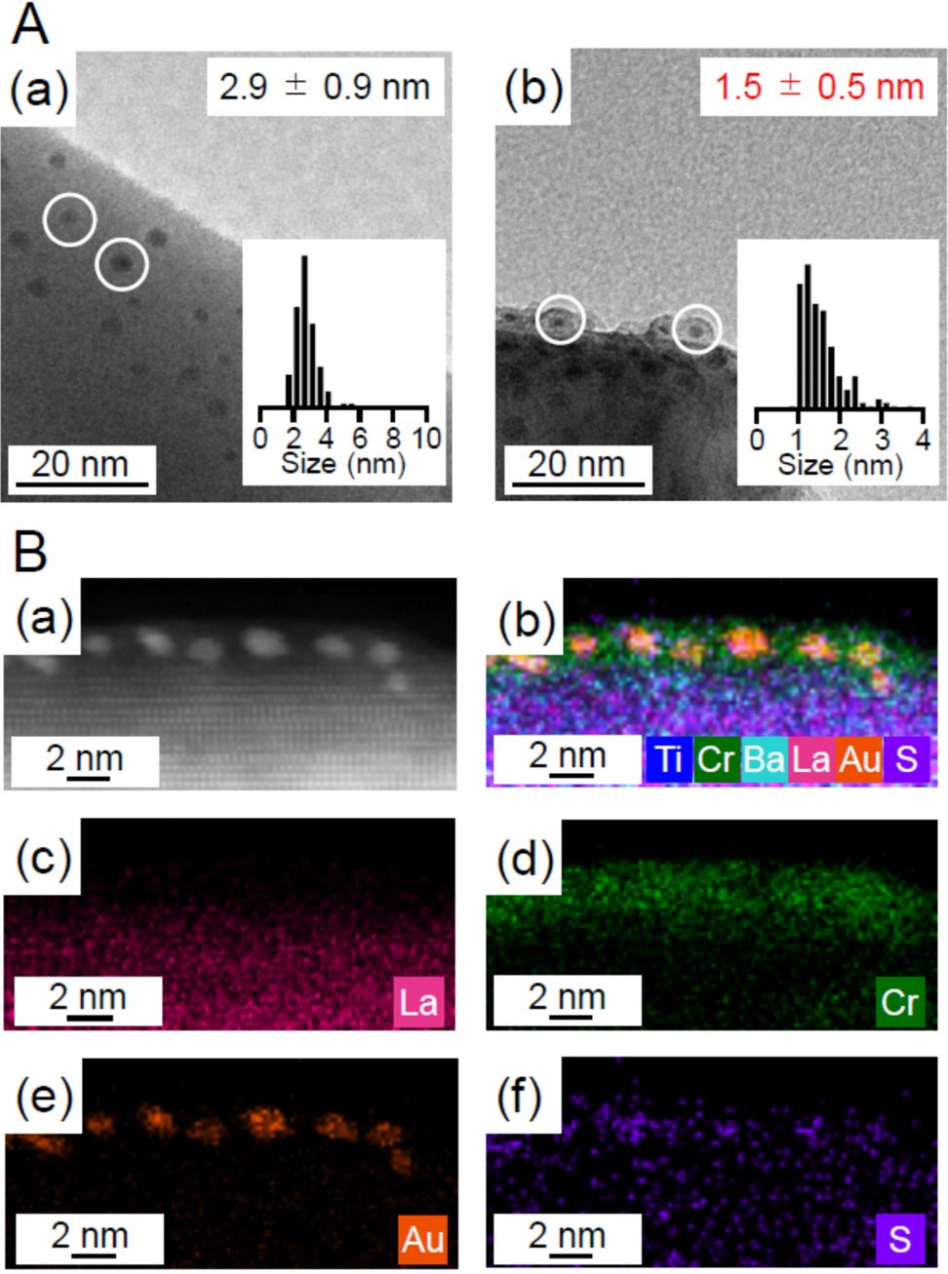
A) (a) TEM images of Au NCs/Cr_2_O_3_/BaLa_4_Ti_4_O_15_ obtained by calcination at 300 °C. This image was measured 30 min after the calcination. (b) TEM images of Cr_2_O_3_/Au NCs/BaLa_4_Ti_4_O_15_, which was obtained by UV irradiation of the sample just after calcination. B) (a) HAADF‐STEM image of Cr_2_O_3_/Au NCs/BaLa_4_Ti_4_O_15_ and (b–f) EDX elemental mapping obtained by HAADF‐STEM image: (b) Ti, Cr, Ba, La, Au, and S, (c) only La, (d) only Cr, (e) only Au, and (f) only S.

The Au L_3_‐edge FT‐EXAFS (Figure S15) and diffuse reflectance spectra (Figure S21) of a series of samples indicate that the Au NCs change their geometric and electronic structures following ligand elimination, similar to the case of Au_25_(PET, *p*‐MBA)_18_/BaLa_4_Ti_4_O_15_.^[43]^ Figure 9 A(a) presents a TEM image of the sample after calcination at 300 °C, revealing the presence of particles with an average size of 2.9±0.9 nm. There are two possible explanations for this finding: 1) in Au_25_(PET, *p*‐MBA)_18_/Cr(OH)_3_/BaLa_4_Ti_4_O_15_, significant aggregation of Au_25_ occurs upon calcination with ligand removal and 2) in Au_25_(PET, *p*‐MBA)_18_/Cr(OH)_3_/BaLa_4_Ti_4_O_15_, the aggregation of Au_25_ is also relatively suppressed during calcination but starts to occur soon thereafter.

In order to clarify the reason for the observed 2.9±0.9 nm particles, we initiated UV‐light irradiation within a few minutes after calcination. The average particle size of the Au NCs was suppressed to 1.5±0.5 nm (Figure 9 A(b)). According to HAADF‐STEM EDX elemental mapping, these Au NCs were embedded in the Cr_2_O_3_ film (Figure 9 B and S22 and Table S2). These results indicate that the aggregation of Au_25_ is relatively suppressed upon heating, even during the calcination of Au_25_(PET, *p*‐MBA)_18_/Cr(OH)_3_/BaLa_4_Ti_4_O_15_; however, significant aggregation of Au_25_ starts soon afterwards.

**Figure 10:**
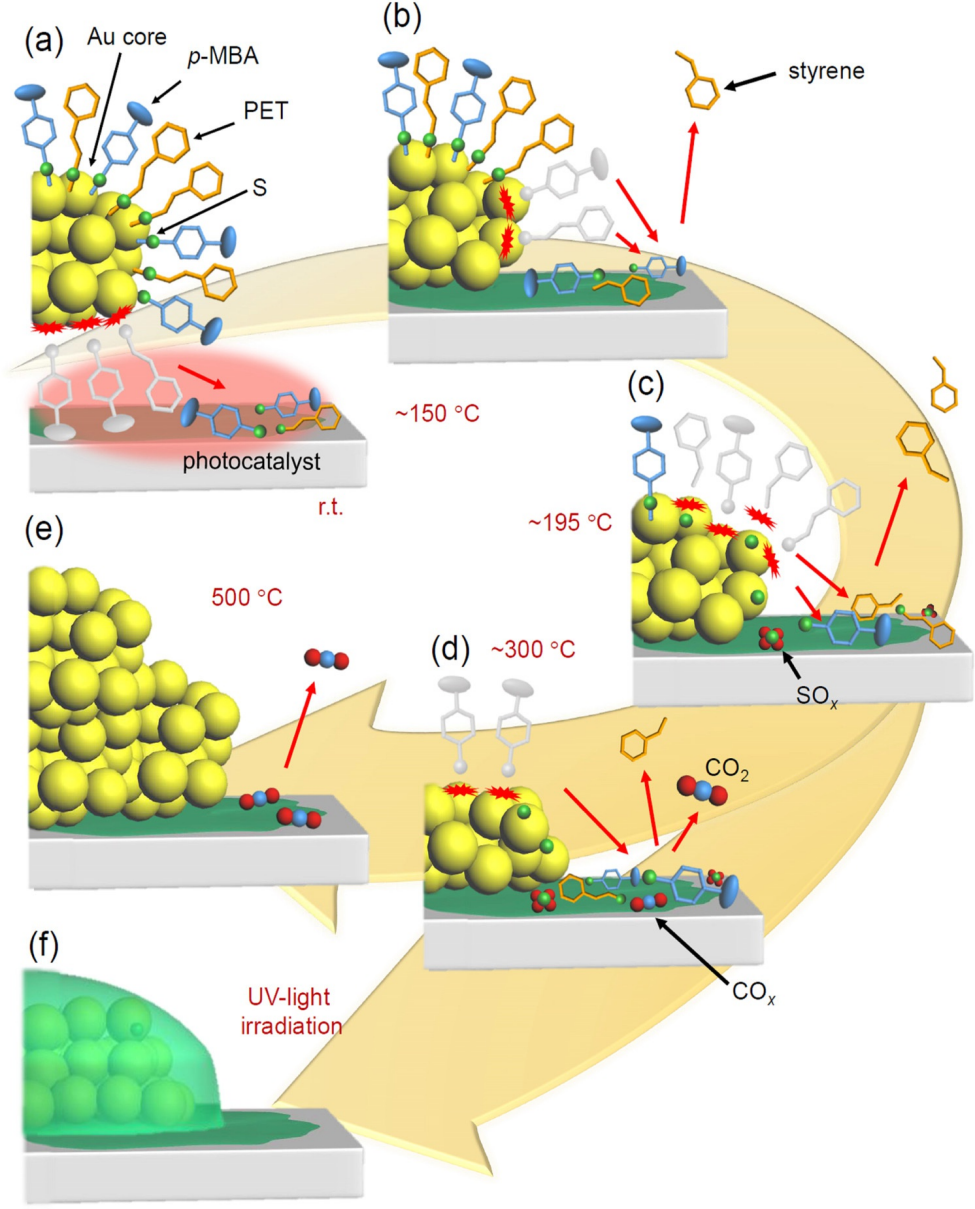
Proposed phenomenon occurring at each temperature and by UV‐light irradiation in Au_25_(PET, *p*‐MBA)_18_/Cr(OH)_3_/BaLa_4_Ti_4_O_15_; at a) room temperature, b) ca. 150 °C, c) ca. 195 °C, d) ca. 300 °C, e) 500 °C, and f) after UV‐light irradiation.

Based on the above results, the behavior of Au_25_(PET, *p*‐ MBA)_18_/Cr(OH)_3_/BaLa_4_Ti_4_O_15_ during calcination and light irradiation can be described as follows (Figure 10):

1. In Au_25_(PET, *p*‐MBA)_18_/Cr(OH)_3_/BaLa_4_Ti_4_O_15_, ligand transfer from Au_25_(PET, *p*‐MBA)_18_ to Cr(OH)_3_/BaLa_4_Ti_4_O_15_ occurs at room temperature (Figure 10(a)). Upon heating, the overall phenomenon is similar to that for Au_25_(PET, *p*‐MBA)_18_/BaLa_4_Ti_4_O_15_. However, it differs from the case of Au_25_(PET, *p*‐MBA)_18_/BaLa_4_Ti_4_O_15_ in that the compound desorption from the support surface occurs before the start of the ligand dissociation from the Au NCs (Figure 10(b)).
2. On Cr_2_O_3_/BaLa_4_Ti_4_O_15_, bare Au_25_ produced by calcination readily aggregates.
3. When Au_25_/Cr_2_O_3_/BaLa_4_Ti_4_O_15_ is irradiated with light, Au NCs are embedded in Cr_2_O_3_ (Figure 10(f)). This phenomenon is assumed to be caused by the transfer of excited electrons generated in the photocatalyst to Au NCs and thereby the reduction of highly oxidized Cr (>3+) to form a deposit over the surface of the Au NCs (Figure S23).

**Figure 11:**
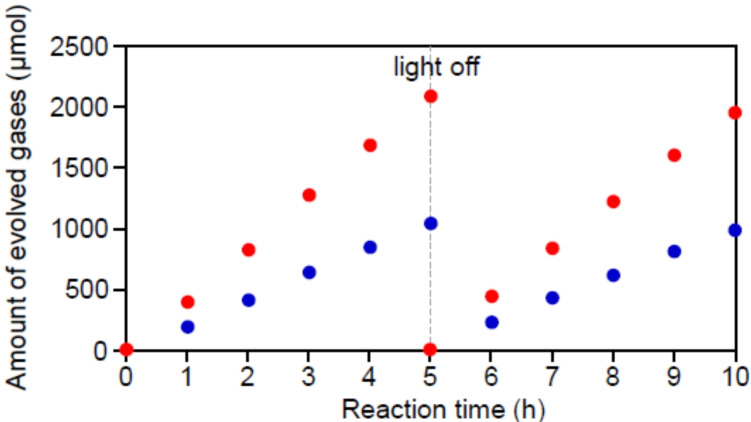
Time course of water‐splitting activity of Cr_2_O_3_/Au NCs/BaLa_4_Ti_4_O_15_ with Au NC particle‐size of 1.5±0.5 nm (Figure 9 A(b)). The red and blue circles represent H_2_ and O_2_, respectively.

Based on this understanding, it is extremely important to reduce the time between calcination and light irradiation as much as possible to create highly functional water‐splitting photocatalysts with fine and stable Au NCs. In fact, the sample in Figure 9 A(b) exhibited a higher water‐splitting activity than the sample with more aggregation (Figure S24 and S25 and Scheme S8). In addition, further aggregation of Au NCs was suppressed even after long‐term exposure to air for this sample, and this sample exhibited high durability during the water‐splitting photocatalysis (Figure 11 and S26). Currently, Cr_2_O_3_ film formation by UV‐light irradiation is performed in pure water (Figure S23). However, the addition of a suitable sacrificial agent to the water would increase the consumption rate of the holes generated by the UV‐light irradiation,[^43^, ^58^, ^59^] thereby allowing the reduction reaction on the surface of the Au NCs, i. e., Cr_2_O_3_ film formation, to occur in a shorter time. It is expected that photocatalysts with even less aggregation of Au NCs can be created in the future by improving the Cr_2_O_3_ film formation method.

## Conclusion

In this study, the calcination mechanisms of Au_25_(PET, *p*‐MBA)_18_/BaLa_4_Ti_4_O_15_ and Au_25_(PET, *p*‐MBA)_18_/Cr(OH)_3_/BaLa_4_Ti_4_O_15_ were investigated, and fine and stable Au NCs‐loaded heterogeneous water‐splitting photocatalysts were created. The following findings were obtained.

1. During the calcination of Au_25_(PET, *p*‐MBA)_18_/BaLa_4_Ti_4_O_15_, the dissociation of S−C and Au−S bonds of Au−PET first starts to occur and then the dissociation of the Au−S bonds of Au−*p*‐MBA starts. The desorbed compounds are then adsorbed onto the support. As the temperature is increased, most of the compounds on the support are desorbed as styrene or CO_2_. At temperatures above 225 °C, the migration of the compound onto the support and the desorption of the compound from the support occur contemporaneously. Although most of the ligands can be removed from the Au_25_ surface by calcination at 300 °C while maintaining the size of Au_25_, some organic compounds and S oxides still remain on the BaLa_4_Ti_4_O_15_ surface even at 500 °C.
2. For the calcination of Au_25_(PET, *p*‐MBA)_18_/Cr(OH)_3_/BaLa_4_Ti_4_O_15_, the overall phenomenon is similar to that of Au_25_(PET, *p*‐MBA)_18_/BaLa_4_Ti_4_O_15_. However, the Au_25_ loaded on Cr_2_O_3_/BaLa_4_Ti_4_O_15_ aggregates significantly with time. To prevent aggregation, light irradiation should be performed soon after calcination to form a Cr_2_O_3_ protective layer on the surface of the Au NCs. When the time between the end of calcination and the start of light irradiation is within a few minutes, the average size of Au NCs in the Cr_2_O_3_ layer can be suppressed to approximately 1.5 nm, and the material maintains a high water‐splitting activity over a long time.

The findings obtained in this study are expected to provide clear design guidelines for the creation of highly functional heterogeneous catalysts using metal NCs, which have been reported thus far.[^12^, ^60^, ^61^]

**Supporting Information**: Experimental section, additional schemes, additional DIP‐MS, XPS, FT‐IR, FT‐EXAFS spectra, TEM image, and photocatalytic activity.

## Conflict of interest

The authors declare no conflict of interest.

## Supporting information

As a service to our authors and readers, this journal provides supporting information supplied by the authors. Such materials are peer reviewed and may be re‐organized for online delivery, but are not copy‐edited or typeset. Technical support issues arising from supporting information (other than missing files) should be addressed to the authors.

Supporting Information
