# Supplementary material for: Creation of High‐Performance Heterogeneous Photocatalysts by Controlling Ligand Desorption and Particle Size of Gold Nanocluster
Source: Angew Chem Int Ed Engl. 2021 Jul 1;60(39):21340–50. doi: 10.1002/anie.202104911 (PMC8518739; doi:10.1002/anie.202104911)
Supplement: Supplementary file 1 — Supporting Information [file ANIE-60-21340-s001.pdf]

Angewandte Chemie
Eine Zeitschrift der Gesellschaft Deutscher Chemiker

Supporting Information

Creation of High-Performance Heterogeneous Photocatalysts by Controlling Ligand Desorption and Particle Size of Gold Nanocluster

*Tokuhisa Kawawaki, Yuki Kataoka, Momoko Hirata, Yuki Akinaga, Ryo Takahata, Kosuke Wakamatsu, Yu Fujiki, Miori Kataoka, Soichi Kikkawa, Abdulrahman S. Alotabi, Sakiat Hossain, D.J. Osborn, Toshiharu Teranishi, Gunther G. Andersson, Gregory F. Metha, Seiji Yamazoe, and Yuichi Negishi**

anie_202104911_sm_miscellaneous_information.pdf

Table of Contents

Experimental Procedures……………………………………………………………………………………………………Page 2

Results and Discussion……………………………………………………………………………………………………Page 5

References……... Page 27

Experimental Procedures

1. Chemicals

All chemicals were obtained commercially and used without further purification. Hydrogen tetrachloroaurate tetrahydrate ($HAuCl_4·4H_2O$) was obtained from Tanaka Kikinzoku. Titanium(IV) tetrabutoxide ($Ti(OC_4H_9)_4$), tetraoctylammonium bromide (TOABr), lanthanum nitrate hexahydrate ($La(NO_3)_3·6H_2O$), barium carbonate ($BaCO_3$), 3-mercaptopropionic acid, 1-buthanethiol, glutathione, bismuth standard solution (100 ppm), and gold standard solution (1000 ppm) were obtained from FUJIFILM Wako Pure Chemical Co.. Tetrahydrofuran (THF), ethanol, methanol, toluene, acetone, acetonitrile, propylene glycol, citric acid, potassium chromate (K_2CrO_4), hydrochloric acid, and nitric acid were obtained from Kanto Chemical Co., Inc. *p*-Mercaptobenzoic acid and 2-phenylethanethiol were obtained from Aldrich. Sodium tetrahydroborate ($NaBH_4$), *trans*-2-[3-(4-*tert*-butylphenyl)-2-methyl-2-propenylidene]malononitrile (DCTB) were obtained from Tokyo Chemical Industry. Co., LTD. Pure Milli-Q water (18.2 MΩ cm) was generated with a Merck Millipore Direct 3 UV system.

2. Synthesis of Metal Nanoclusters

2.1. $[Au_{25}(PET)_{18}]^-$.

$[Au_{25}(PET)_{18}]^-$ was synthesized by the method reported by Jin and co-workers[1] with slight modification. First, 0.75 mmol of $HAuCl_4·4H_2O$ was dissolved in 25 mL of a THF solution containing 0.76 mmol of TOABr. After the reaction mixture was stirred for 15 min, 4.7 mmol of 2-phenylethanethiol was added to the solution. After 15 min of stirring, 5 mL of a cold (0 °C) aqueous solution containing 8.7 mmol of $NaBH_4$ was rapidly added. After 12 h of stirring, THF was evaporated, and the remaining red-brown powder was washed with methanol to remove excess thiol and other byproducts. Finally, pure $[Au_{25}(PET)_{18}]^-$ was extracted from the precipitate of the mixture using acetonitrile (Scheme S3).

2.2. $[Au_{25}(PET, p\text{-MBA})_{18}]^-$.

$[Au_{25}(PET, p\text{-MBA})_{18}]^-$ was prepared by exchanging a part of the ligand of $[Au_{25}(PET)_{18}]^-$ with *p*-MBA (not thiol but thiolate).[2] In the experiment, first, 3 mg of $[Au_{25}(PET)_{18}]^-$ was dissolved in 1 mL of acetone. Then, 3 mg of *p*-mercaptobenzoic acid was added to this solution. The solution was left at room temperature for 2 h. The obtained product was dried by evaporation and then the dried product was washed with a mixture of methanol and water (7:3) to remove excess thiol and other byproducts. This washing operation was repeated at least five times. The obtained $[Au_{25}(PET, p\text{-MBA})_{18}]^-$ with a lower number of *p*-MBA ligands was then reacted again with *p*-mercaptobenzoic acid under the same experimental conditions to increase the number of *p*-MBA ligands in $[Au_{25}(PET, p\text{-MBA})_{18}]^-$.

2.3. $[Au_{25}(PET, 3\text{-MPA})_{18}]^-$.

$[Au_{25}(PET, 3\text{-MPA})_{18}]^-$ was prepared by exchanging a part of the ligand of $[Au_{25}(PET)_{18}]^-$ with 3-MPA (not thiol but thiolate). In the experiment, first, 3 mg of $[Au_{25}(PET)_{18}]^-$ was dissolved in 1 mL of acetone. Then, 2.7 µL of 3-mercaptopropionic acid was added to this solution. The solution was stirred at room temperature for 2 h. The obtained product was dried by evaporation, and then the dried product was washed with a mixture of methanol and water (3:7) to remove excess thiol and other byproducts. This washing operation was repeated at least five times. The obtained $[Au_{25}(PET, 3\text{-MPA})_{18}]^-$ with a lower number of 3-MPA ligands was then reacted again with 3-mercaptopropionic acid under the same experimental conditions to increase the number of 3-MPA ligands in $[Au_{25}(PET, 3\text{-MPA})_{18}]^-$.

2.4. $[Au_{25}(SC4)_{18}]^-$.

First, 0.75 mmol of $HAuCl_4·4H_2O$ was dissolved in 25 mL of THF solution containing 0.76 mmol of TOABr. After the reaction mixture was stirred for 15 min, 4.9 mmol of 1-buthanethiol was added to the solution. After 30 min of stirring, 5 mL of a cold (0 °C) aqueous solution containing 8.7 mmol of $NaBH_4$ was rapidly added. After 10 h of stirring, THF was removed by rotary evaporator. Excess $NaBH_4$ was washed with water two times and then washed with mixture of water and methanol (4:1, 1:1, and finally 1:4). After that, the product was extracted by methanol and the solvent was removed from the sample by rotary evaporator. Finally, $[Au_{25}(SC4)_{18}]^-$ was extracted from the precipitate using toluene.

2.5. $[Au_{25}(SC4, p\text{-MBA})_{18}]^-$.

$[Au_{25}(SC4, p\text{-MBA})_{18}]^-$ was prepared by exchanging a part of the ligand of $[Au_{25}(SC4)_{18}]^-$ with *p*-MBA. In the experiment, first, 3 mg of $[Au_{25}(SC4)_{18}]^-$ was dissolved in 1 mL of acetone. Then, 3 mg of *p*-mercaptobenzoic acid was added to this solution. The solution was left at room temperature for 2 h. The obtained product was dried by evaporation and then the dried product

was washed with a mixture of methanol and water (7:3) to remove excess thiol and other byproducts. This washing operation was repeated at least five times. The obtained $[Au_{25}(SC4, p\text{-MBA})_{18}]^-$ with a lower number of *p*-MBA ligands was then reacted again with *p*-mercaptobenzoic acid under the same experimental conditions to increase the number of *p*-MBA ligands in $[Au_{25}(SC4, p\text{-MBA})_{18}]^-$.

3. Preparation of Heterogeneous Catalysts

The overall flowing of the preparation of heterogeneous catalysts is depicted in Scheme 2. In the followings, the detail of the experiments is described.

3.1. $BaLa_4Ti_4O_{15}$

$BaLa_4Ti_4O_{15}$, which shows high water-splitting activity by loading Au particles, was used as the water-splitting photocatalyst. The $BaLa_4Ti_4O_{15}$ photocatalyst was prepared by a polymerized complex method.[3] In brief, 6.06 g (17.8 mmol) of $Ti(OC_4H_9)_4$ and 60.9 g (800 mmol) of propylene glycol were added to 19 mL of ethanol, and the solution was then heated to 70 °C. Subsequently, 38.4 g (200 mmol) of citric acid, 7.71 g (17.8 mmol) of $La(NO_3)_3$, and 0.878 g (4.45 mmol) of $BaCO_3$ were sequentially added, and the solution was heated at 120–130 °C for 5 h. The obtained gray powder was transferred to an electric furnace and heated at 500 °C and then at 1100 °C. About 5.0 g of $BaLa_4Ti_4O_{15}$ was obtained (Scheme S4).

3.2. $Cr(OH)_3/BaLa_4Ti_4O_{15}$

A $Cr(OH)_3$ layer was loaded on $BaLa_4Ti_4O_{15}$ by photodeposition.[4] In this process, $BaLa_4Ti_4O_{15}$ (650 mg) was added to an aqueous K_2CrO_4 solution (350 mL) in a quartz cell. The mixing ratio of K_2CrO_4 to $BaLa_4Ti_4O_{15}$ was fixed at 0.3 wt% Cr. After removing dissolved air by argon (Ar) bubbling, the suspension was irradiated with a high-pressure mercury (Hg) lamp (400 W) under an Ar flow of 30 mL/min at 25 °C for 1.5 h. The actual amounts of Cr loaded on $BaLa_4Ti_4O_{15}$ were determined by inductively coupled plasma mass spectrometry (ICP-MS) of the aqueous solution after mixing. The solid $Cr(OH)_3/BaLa_4Ti_4O_{15}$ was collected by centrifugation, washed with water three times, and dried by evaporation. It is noted that the chemical composition of $Cr(OH)_3$ layer was elucidated in this study (Figure S17 and S18), thus $Cr(OH)_3/BaLa_4Ti_4O_{15}$ has been described as $Cr_2O_3/BaLa_4Ti_4O_{15}$ in our previous papers.[2,4]

3.3. $Au_{25}(PET, p\text{-MBA})_{18}/BaLa_4Ti_4O_{15}$ and $Au_{25}(PET, p\text{-MBA})_{18}/Cr(OH)_3/BaLa_4Ti_4O_{15}$

Acetone solutions containing $[Au_{25}(PET, p\text{-MBA})_{18}]^-$ were prepared and their concentrations were accurately determined by ICP-MS. Then, the solution was mixed with 600 mg of the synthesized photocatalyst ($BaLa_4Ti_4O_{15}$ or $Cr(OH)_3/BaLa_4Ti_4O_{15}$) and stirred for 1 hour at room temperature.[2] The mixing ratio of $[Au_{25}(PET, p\text{-MBA})_{18}]^-$ to $BaLa_4Ti_4O_{15}$ or $Cr(OH)_3/BaLa_4Ti_4O_{15}$ was fixed at 0.100 wt% Au because this gives a photocatalyst with high activity.[5] The amount of $[Au_{25}(PET, p\text{-MBA})_{18}]^-$ actually adsorbed on $BaLa_4Ti_4O_{15}$ or $Cr(OH)_3/BaLa_4Ti_4O_{15}$ was estimated by ICP-MS. The obtained product was separated from solution and dried by evaporation.

3.4. $Au_{25}/BaLa_4Ti_4O_{15}$.

The obtained $Au_{25}(PET, p\text{-MBA})_{18}/BaLa_4Ti_4O_{15}$ was calcined by an electric furnace under the reduced pressure (~1.0 × 10^{-1} Pa; Scheme S5). The temperature was increased with a heating rate of 7 °C/min and kept at 300 °C for 80 min.

3.5. $Cr_2O_3/Au_{25}/BaLa_4Ti_4O_{15}$.

The obtained $Au_{25}(PET, p\text{-MBA})_{18}/Cr(OH)_3/BaLa_4Ti_4O_{15}$ was calcined by an electric furnace under the reduced pressure (>1.0 × 10^{-1} Pa; Scheme S5). The temperature was increased with a heating rate of 7 °C/min and kept at 300 °C for 80 min. The obtained $Au_{25}/Cr_2O_3/BaLa_4Ti_4O_{15}$ was dispersed into water, which was filled in an experimental apparatus for measuring water-splitting activity (Scheme S8). The water including $Au_{25}/Cr_2O_3/BaLa_4Ti_4O_{15}$ was irradiated by UV-light with Hg lamp (400 W) for 1 h to induce the coverage of Au_{25} by Cr_2O_3 shell ($Cr_2O_3/Au_{25}/BaLa_4Ti_4O_{15}$).[2] To suppress the aggregation of Au_{25}, the light irradiation was started within a few minutes after dispersing the $Au_{25}/Cr_2O_3/BaLa_4Ti_4O_{15}$ into water.

4. Characterization

The matrix assisted laser desorption/ionization (MALDI) mass spectra were recorded with a spiral time-of-flight mass spectrometer (JMSS3000, JEOL, Tokyo, Japan) equipped with a semiconductor laser (λ = 349 nm). DCTB was used as the MALDI matrix. To minimize NC dissociation induced by laser irradiation, the NC-to-matrix ratio was fixed at 1:1000.

The diffuse reflection (DR) spectra were acquired at ambient temperature with a V-670 spectrometer (JASCO, Tokyo, Japan). The wavelength-dependent optical data ($I(w)$) were converted to energy-dependent data ($I(E)$) by the following equation that conserved the integrated spectral areas: $I(E) = I(w)/|\partial E/\partial w| \propto I(w) \times w^2$.

ICP-MS was performed with an Agilent 7500c spectrometer (Agilent Technologies, Tokyo, Japan). Bismuth was used as the internal standard. The ICP-MS measurements were performed for the supernatant obtained after mixing $[Au_{25}(PET, p\text{-}MBA)_{18}]^-$ with the photocatalyst ($BaLa_4Ti_4O_{15}$ or $Cr_2O_3/BaLa_4Ti_4O_{15}$) to estimate the unadsorbed Au content. The adsorption efficiency in each experiment was estimated on the basis of this value.

Direct insertion probe-mass spectrometry (DIP-MS) was performed with JMS-Q1500GC analyzer (JEOL, Tokyo, Japan). The sample was introduced into a glass tube with a diameter of approximately 1.6 mm and heated with a ratio of 1 °C/min from 80 to 500 °C under reduced pressure (~1.0×10^{-4} Pa). The desorbed ligand was directly inserted to the mass spectrometer where each compound was ionized by electron impact (Scheme S6). The peaks with m/z = 18 (water), 28 (carbon mono-oxide or nitrogen molecule), 207, and 281 (derived from column or grease) were observed even when the sample was not loaded.

The Au L_3-edge X-ray absorption fine structure (XAFS) measurements were performed at beamlines BL01B1 of the SPring-8 facility of the Japan Synchrotron Radiation Research Institute (proposal numbers 2018B0919, 2018B1422, 2020A0695, 2020A0715 and 2020A1410). The incident X-ray beam was monochromatized by a silicon (Si) (111) double-crystal monochromator. The XAFS spectra of Au foil (Au L_3-edge) as reference was recorded in transmission mode using ionization chambers. The Au L_3-edge XAFS spectra of the photocatalyst were measured in fluorescence mode using a 19-element Ge solid-state detector at room temperature. The X-ray energies for the Au L_3-edge were calibrated using Au foil. The X-ray absorption near edge structure (XANES) and extended X-ray absorption fine structure (EXAFS) spectra were analyzed using the xTunes program[6] as follows. The χ spectra were extracted by subtracting the atomic absorption background by cubic spline interpolation and normalized to the edge height. The normalized data were used as the XANES spectra. The k^3-weighted χ spectra in the k range 3.0–12.0 $Å^{-1}$ for the Au L_3-edge was Fourier transformed into r space.

The Cr $L_{2,3}$-edge XAFS measurements were performed at the soft X-ray beamline in the Australian Synchrotron. Samples were dispersed in ethanol and drop-coated onto a Si wafer prior to measurement.

Fourier transform infrared (FT-IR) spectrum of the product was obtained using the attenuated total reflectance (ATR) method. FT-IR ATR spectra were recorded in the region between 400 and 4000 cm^{-1} using a JASCO FT/IR–4600–ATR–PRO ONE spectrometer equipped with a DLATGS detector as the average of 100 scans.

The Au 4f and Cr 2p X-ray photoelectron spectroscopy (XPS) spectra were obtained using a SPECS Phoibos 100 hemispherical electron analyzer from SPECS in conjunction with a non-monochromatic X-ray source using the Mg Kα line ($h\nu$ =1253.6 eV).[7] A 10 mL drop of each sample was deposited onto a clean 66 mm Si wafer and dried in air. Each sample was then fixed by double-sided copper tape onto a Au-plated holder which were affixed onto each plate for XPS analysis.

The S 2p XPS spectra were collected by using a JPS-9010MC electron spectrometer (JEOL, Tokyo, Japan) at a base pressure of ~2×10^{-8} Torr. X-rays from the Mg-Kα line (1253.6 eV) were used for excitation. The spectra were calibrated with the peak energies of C 1s (284.6 eV).

The TEM images were recorded with a JEM-2100 electron microscope (JEOL, Tokyo, Japan) operating at 200 kV, typically using magnification of 400 000–600 000.

The high-angle annular dark field scanning TEM (HAADF-STEM) images were obtained by ultra-high-resolution transmission electron microscope (The FEI Titan Themis 80–200) operating at 200 kV, with a beam convergence semi angle of 25 mrad and HAADF collection angle from 56–200 mrad. Elemental maps were acquired using a super X detector and low-background sample holder.

5. Measurement of the Photocatalytic Water-splitting Activity

The photocatalytic water-splitting reaction was performed at room temperature using an experimental apparatus built in-house consisting of a high-pressure Hg lamp (400 W) and a quartz cell (Scheme S7).[5] The reaction was performed with a flowing Ar gas at the rate of 30 mL/min. Before the measurements, the reaction solution containing the prepared photocatalyst (50 mg) in water (350 mL) was purged with Ar gas for 1 h to ensure complete removal of air from the reaction vessel. The evolved gases were analyzed by Shimadzu GC-8A gas chromatograph equipped with a thermal conductivity detector and a MS-5A column (Shimadzu, Kyoto, Japan).

Results and Discussion

1. Table

Table S1. Assignments of Peaks Observed in DIP-MS Spectra

m/*z*	Chemical formula	Structural formula	Parent molecule
29	C_2H_5	H_3C-CH_2	SC4
41	C_3H_5	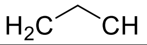	SC4
44	CO_2	O=C=O	PET, *p*-MBA, and SC4, 3-MPA
57	C_4H_9	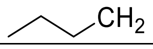	SC4
65	C_5H_5	Unknown	PET and *p*-MBA
77	C_6H_5	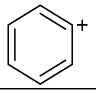	PET and *p*-MBA
78	C_6H_6	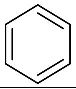	PET
91	$C_6H_5CH_2$	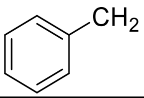	PET
104	$C_6H_5C_2H_3$	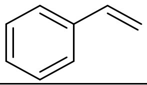	PET
105	$C_6H_5C_2H_4$ or SC_2H_4COOH	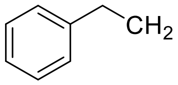 or 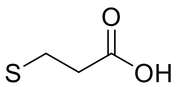	PET or 3-MPA
109	C_6H_5S	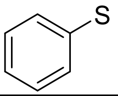	*p*-MBA
137	SC_6H_5CHO	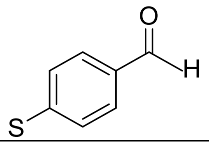	*p*-MBA
154	HSC_6H_5COOH	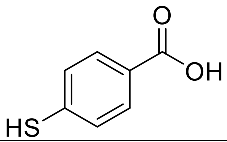	*p*-MBA
186	Unknown	Unknown	*p*-MBA−SC4
242	$C_4H_9S_2C_6H_4COOH$	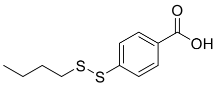 or 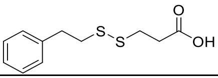	*p*-MBA−SC4 or PET−3-MPA

254	$NC_{17}H_{36}$	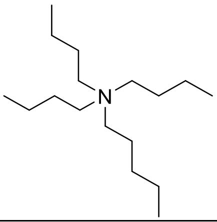	TOA^+
274	$(C_6H_5C_2H_4S)_2$	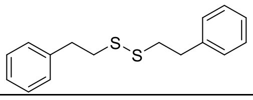	PET
290	$C_6H_5C_2H_4S_2C_6H_4COOH$	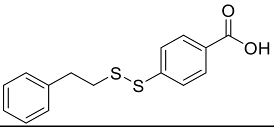	PET−*p*-MBA

Table S2. Analysis of EDX spectrum shown in Figure S22.

Z	Element	Family	Atomic Fraction (%)	Atomic Error (%)	Mass Fraction (%)	Mass Error (%)	Fit error (%)
8	O	K	61.60	4.65	24.96	1.24	0.44
16	S	K	0.42	0.09	0.34	0.07	5.09
22	Ti	K	11.42	1.74	13.84	1.96	0.41
24	Cr	K	15.68	2.40	20.65	2.93	0.20
56	Ba	L	0.05	0.02	0.18	0.06	30.07
57	La	L	9.46	1.25	33.28	3.96	0.32
79	Au	L	1.35	0.18	6.74	0.82	0.74

2. Additional Schemes

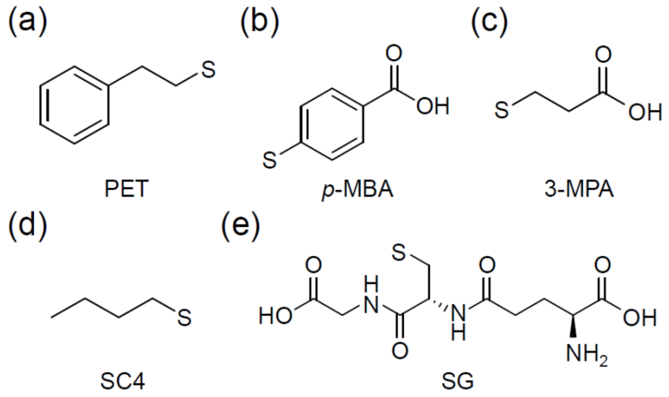

Scheme S1. Thiolates used in this study; (a) PET, (b) *p*-MBA, (c) 3-MPA, (d) SC4, and (e) gluthathionate (SG).

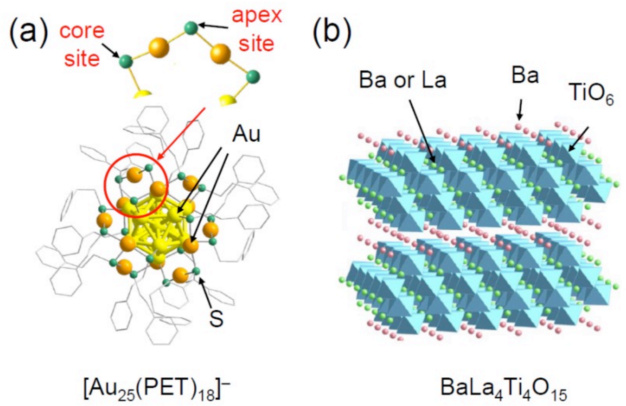

Scheme S2. Geometrical structures of (a) $[Au_{25}(PET)_{18}]^-$ and (b) $BaLa_4Ti_4O_{15}$. In (a), two kinds of S exist (core site and apex site).

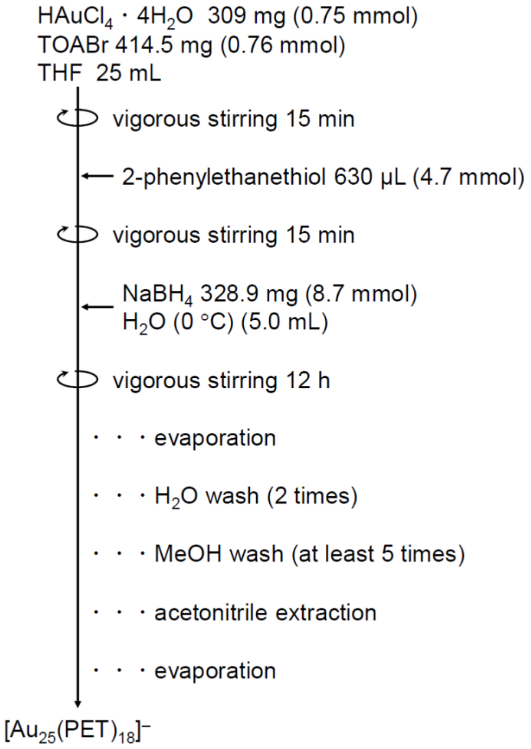

Scheme S3. Protocol used for the synthesis of $[Au_{25}(PET)_{18}]^-$.

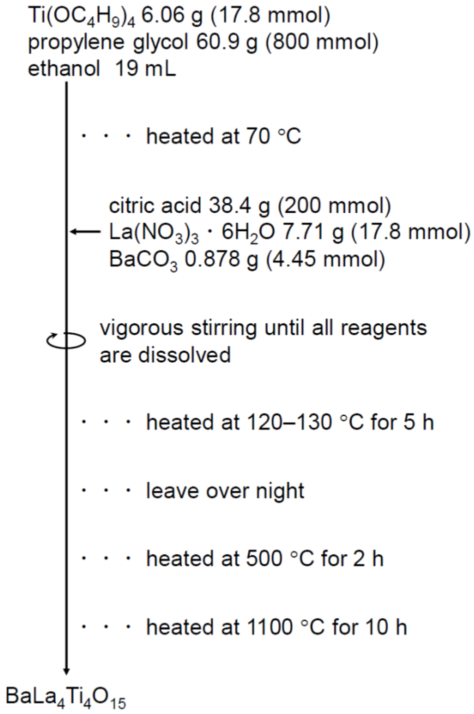

Scheme S4. Protocol used for the synthesis of $BaLa_4Ti_4O_{15}$.

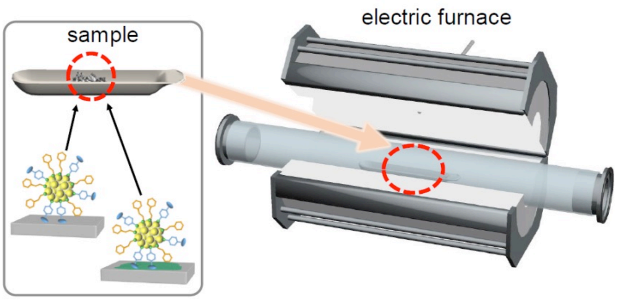

Scheme S5. Schematic of the system used for the calcination of the catalysts.

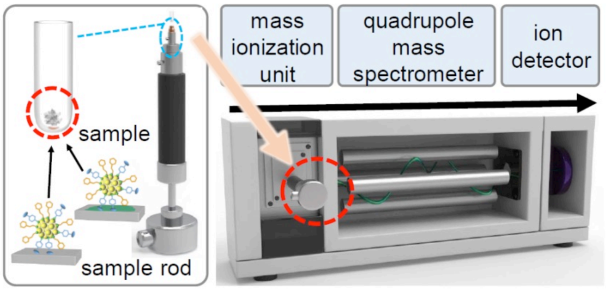

Scheme S6. Schematic of the experiment of DIP-MS.

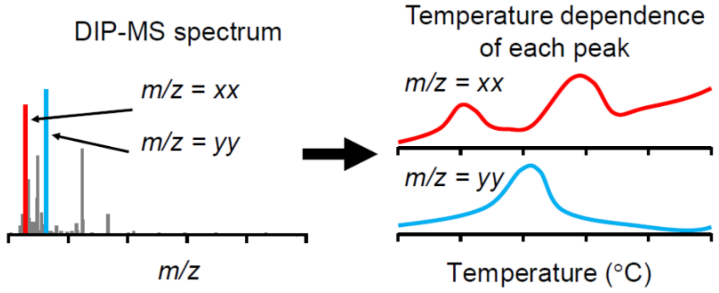

Scheme S7. Schematic of the relation between DIP-MS spectrum (*e. g.* Figure 1A) and the temperature dependence of each mass peak (*e. g.* Figure 1B).

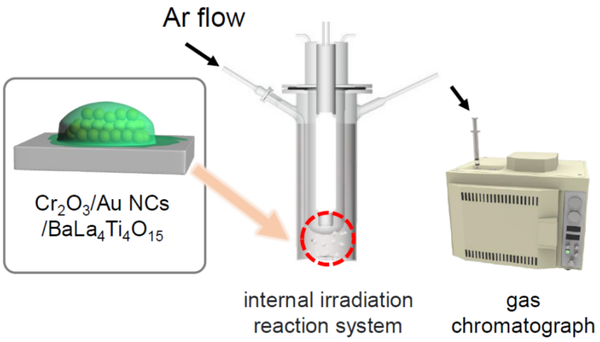

Scheme S8. Schematic of the measurement of the photocatalytic water-splitting activity of Cr_2O_3/Au NCs/ $BaLa_4Ti_4O_{15}$.

4. Additional Figures

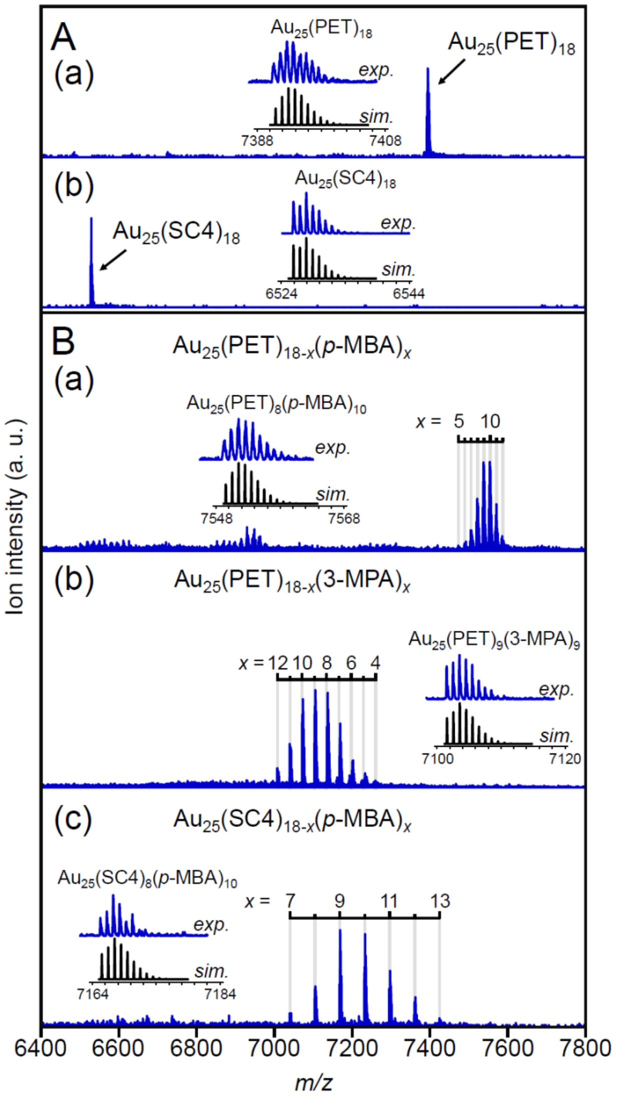

Figure S1. (A) MALDI mass spectra of (a) $[Au_{25}(PET)_{18}]^-$ and (b) $[Au_{25}(SC4)_{18}]^-$. (B) MALDI mass spectra of the ligand-exchanged products: (a) $[Au_{25}(PET, p\text{-}MBA)_{18}]^-$, (b) $[Au_{25}(PET, 3\text{-}MPA)_{18}]^-$, and (c) $[Au_{25}(SC4, p\text{-}MBA)_{18}]^-$.

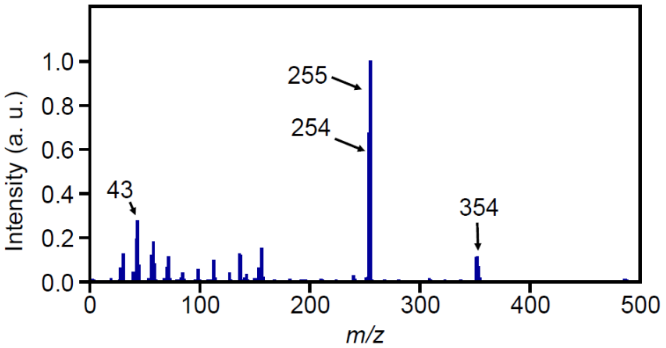

Figure S2. DIP-MS spectrum of TOABr. This spectrum demonstrates that the peak at around *m*/*z* = 254 observed in Figure 1A is due to the EI-fragment of TOA^+ (*m*/*z* = 467).

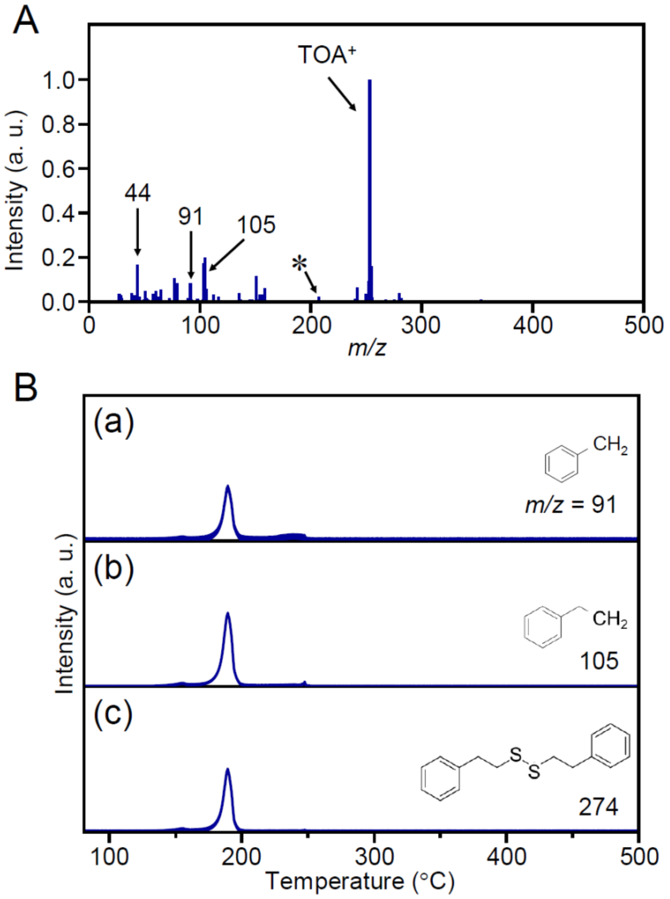

Figure S3. (A) DIP-MS spectrum of the compounds desorbed from $Au_{25}(PET)_{18}$ in the range from 80 °C to 500 °C. In this spectrum, the peak with asterisk (*) is not due to the sample but due to the compounds deposited in the apparatus (see section 1.4). The peak at *m*/*z* = 44 is assigned to the final product by calcination, carbon dioxide (CO_2). (B) Temperature dependence of each mass peak: *m*/*z* = (a) 91, (b) 105, and (c) 274. This result confirmed that the peaks at *m*/*z* = 91, 105, and 274 are originated from PET.

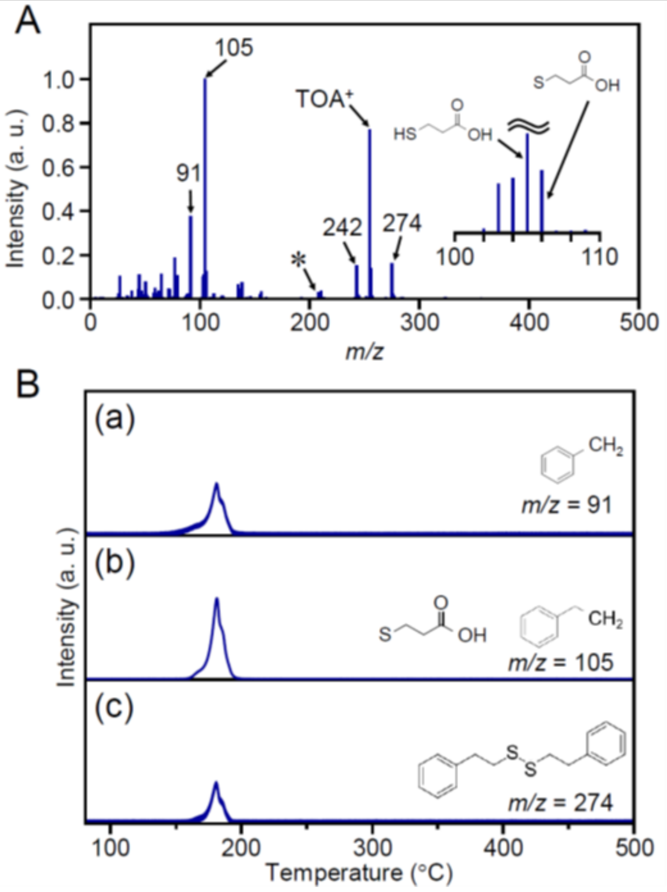

Figure S4. (A) DIP-MS spectrum of the compounds desorbed from Au_{25}(PET, 3-MPA)$_{18}$ in the range from 80 °C to 500 °C. In this spectrum, the peak with asterisk (*) is not due to the sample but due to the compounds deposited in the apparatus (see section 1.4). The peak at *m*/*z* = 242 is assigned to the compound formed by the connection between PET and 3-MPA, PET−3-MPA. The inset shows the enlarged spectrum at around *m*/*z* = 105. The peak assignable to 3-MPA (not thiolate but thiol) appears at *m*/*z* = 106, implying that 3-MPA is also detached in the form of thiol together with the form of thiolate. (B) Temperature dependence of each mass peak: *m*/*z* = (a) 91, (b) 105, and (c) 274. This result confirmed that the peaks at *m*/*z* = 91, 105, and 274 are originated from PET.

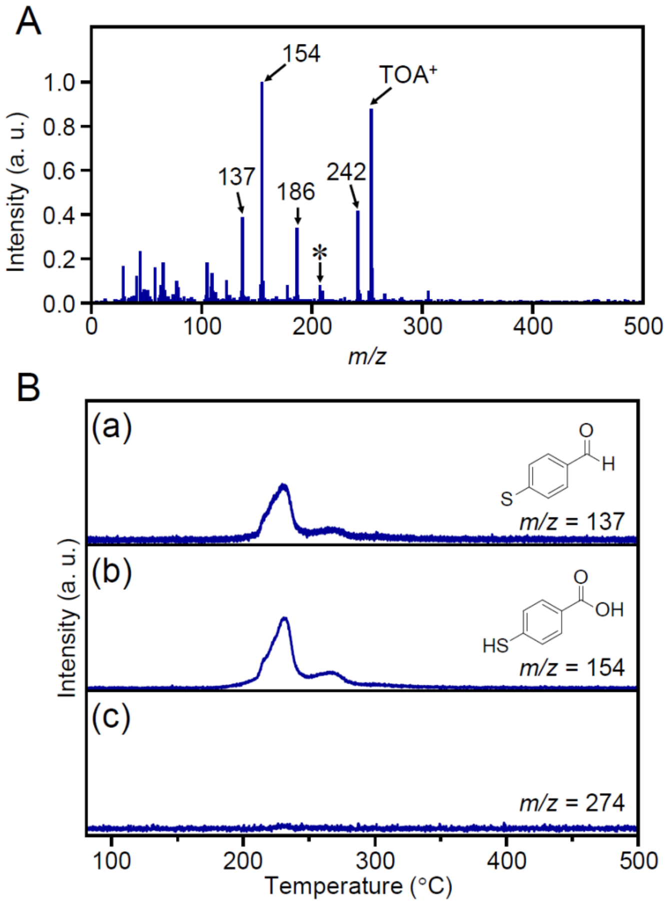

Figure S5. (A) DIP-MS spectrum of the compounds desorbed from Au_{25}(SC4, *p*-MBA)$_{18}$ in the range from 80 °C to 500 °C. In this spectrum, the peak with asterisk (*) is not due to the sample but due to the compounds deposited in the apparatus (see section 1.4). The peak at *m*/*z* = 242 is assigned to the compound formed by the connection of SC4 and *p*-MBA, SC4−*p*-MBA. The peak at *m*/*z* = 186 was difficult to assign. However, since this peak was not observed in the DIP-MS spectrum of Au_{25}(PET, *p*-MBA)$_{18}$, this peak seems to be originated from SC4. (B) Temperature dependence of each mass peak: *m*/*z* = (a) 137 and (b) 154. These results confirmed that the peaks at *m*/*z* = 137 and 154 are originated from *p*-MBA. In this spectrum, the peaks at *m*/*z* = 91, 105, and 274 (c) were not observed, indicating that the peaks at *m*/*z* = 91, 105, and 274 are originated from PET.

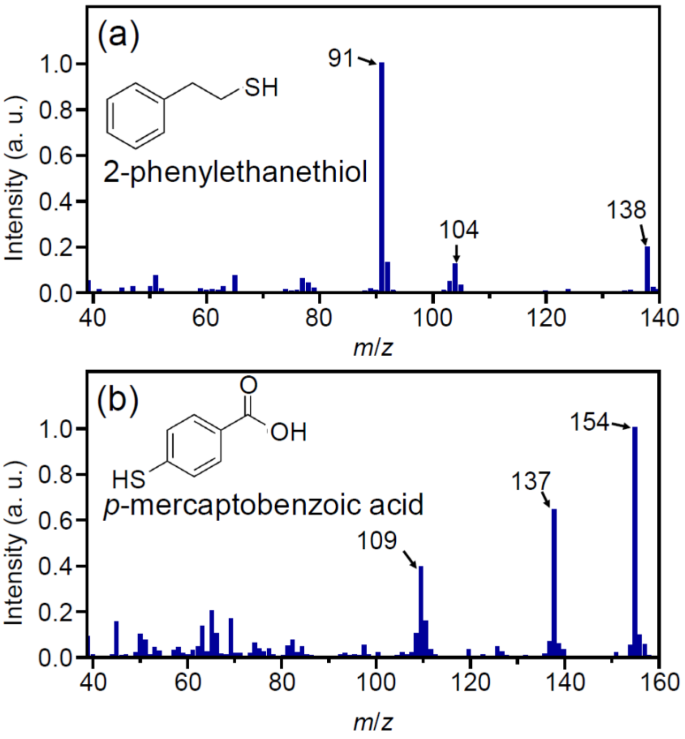

Figure S6. EI-MS spectra of (a) 2-phenylethanethiol and (b) *p*-mercaptobenzoic acid. These mass spectra were reproduced from the data base of GC-MS (GCMS-QP2010 SE). Figure (a) shows that peaks at *m*/*z* = 91 and 104 can be caused by EI of 2-phenylethanethiolate, but peak at *m*/*z* = 105 could not be, indicating that the peak at *m*/*z* = 105, which was observed in Figure 1A, is due to the thermal dissociation of S−C bond in Au−PET. Figure (b) shows that peak at *m*/*z* = 137 can be caused by EI of *p*-mercaptobenzoic acid.

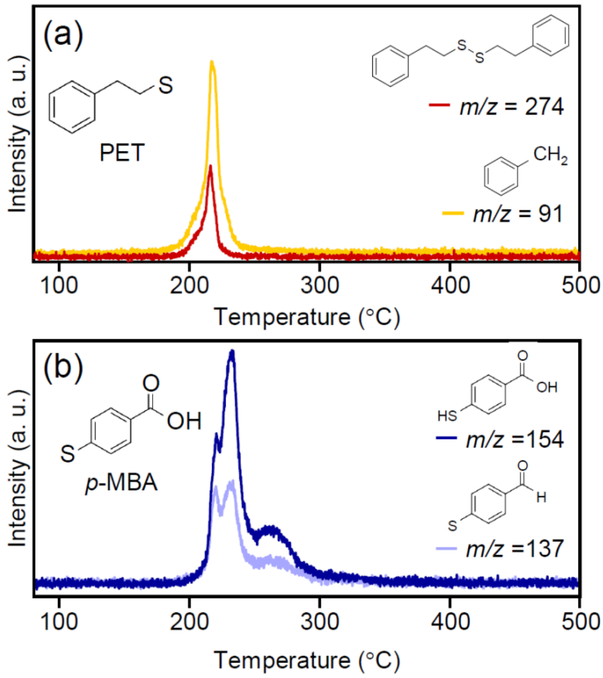

Figure S7. Comparison of the temperature dependence of each peak observed in the DIP-MS spectrum of Au_{25}(PET, *p*-MBA)$_{18}$: (a) peaks at *m*/*z* = 91 and 274; (b) peaks at *m*/*z* = 137 and 154. Figure (a) verifies that the peaks at *m*/*z* = 91 is EI-fragments of $(PET)_2$ (*m*/*z* = 274). Similarly, Figure (b) verifies that the peaks at *m*/*z* = 137 is EI-fragments of *p*-MBA (*m*/*z* = 154; not thiolate but thiol).

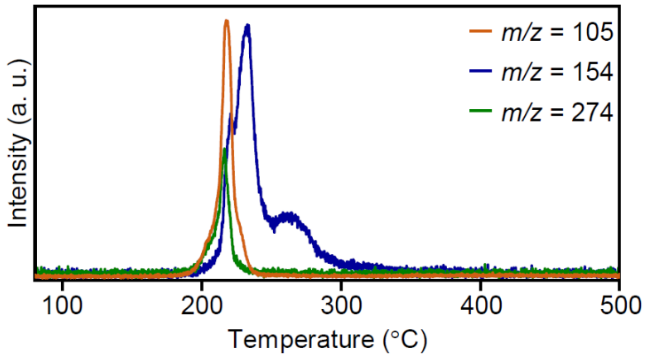

Figure S8. Comparison of the temperature dependence of each peak observed in the DIP-MS spectrum of Au_{25}(PET, *p*-MBA)$_{18}$: PE (*m*/*z* = 105), $(PET)_2$ (*m*/*z* = 274), and *p*-MBA (*m*/*z* = 154; not thiolate but thiol). This result indicates that in the calcination of Au_{25}(PET, *p*-MBA)$_{18}$, S−C and Au−S bonds in Au−PET first start to dissociate (at ~195 °C) and then Au−S bond in Au−*p*-MBA starts to dissociate (at ~225 °C).

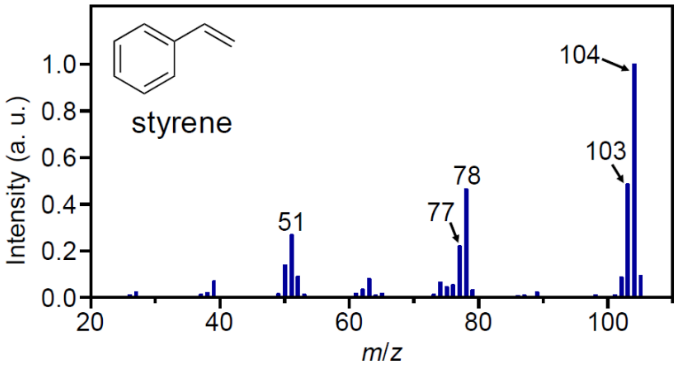

Figure S9. EI-MS spectra of styrene. This mass spectrum was reproduced from the data base of GC-MS (GCMS-QP2010 SE). This spectrum demonstrates that the peak at *m*/*z* = 78 observed in Figure 3A and 8A is due to the EI-fragment of styrene (*m*/*z* = 104).

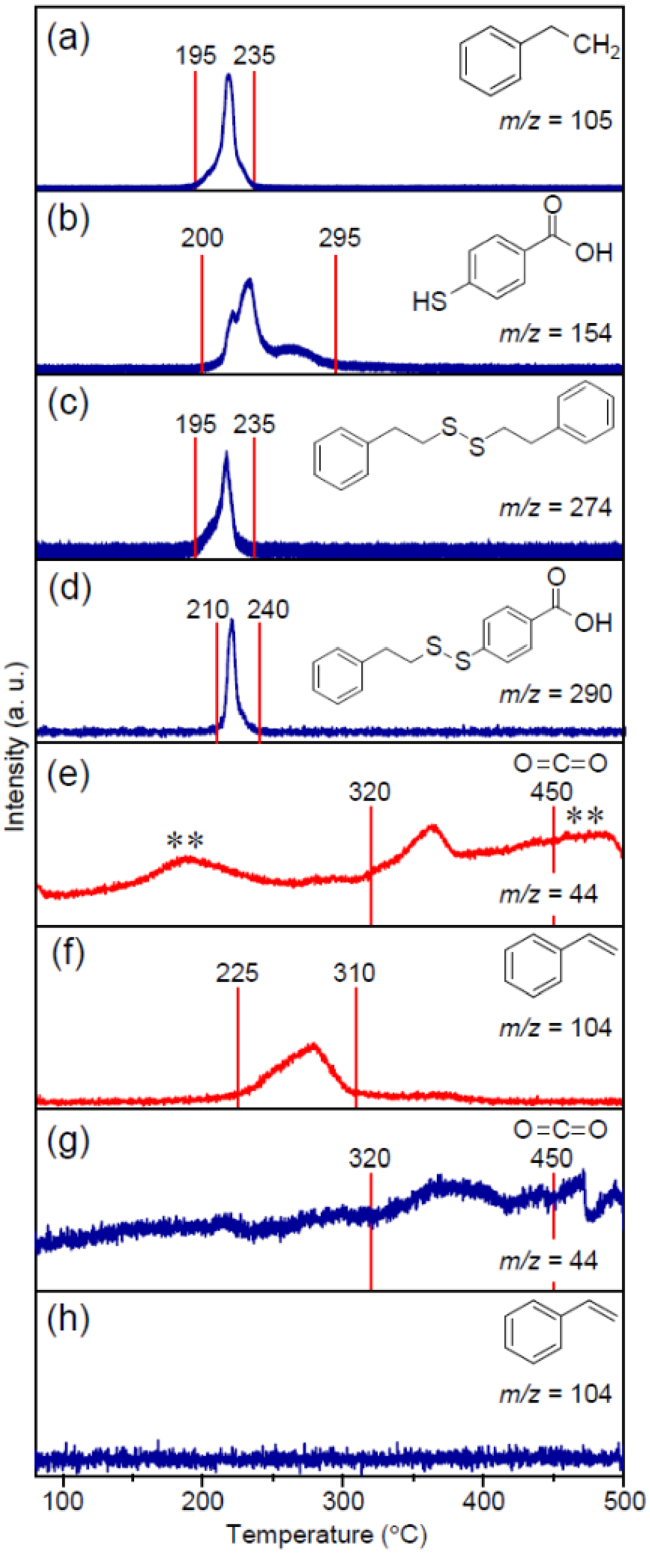

Figure S10. Comparison of the temperature dependence of each peak: (a) PE, (b) *p*-MBA (not thiolate but thiol), (c) $(PET)_2$, and (d) PET−*p*-MBA observed in the calcination of Au_{25}(PET, *p*-MBA)$_{18}$, whereas (e, g) CO_2 and (f, h) styrene observed in the calcination of (e, f) Au_{25}(PET, *p*-MBA)$_{18}$/$BaLa_4Ti_4O_{15}$ and (g, h) *p*-MBA/$BaLa_4Ti_4O_{15}$.In (e−h), the peak start to appear at the higher temperatures than those of PE, *p*-MBA, and $(PET)_2$ ((a)−(d)), strongly implies that PE, *p*-MBA, and $(PET)_2$, which are fragmentated from Au_{25}(PET, *p*-MBA)$_{18}$, once adsorbed on the $BaLa_4Ti_4O_{15}$ and then desorbed from the surface of $BaLa_4Ti_4O_{15}$ in the form of styrene or CO_2.

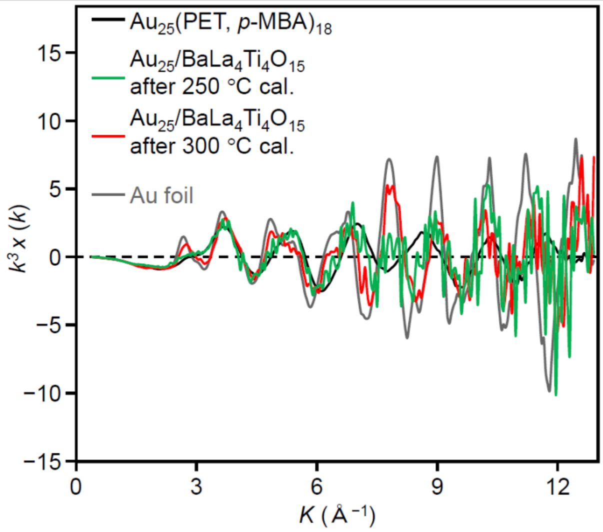

Figure S11. Au L_3-edge EXAFS spectra of Au_{25}(PET, *p*-MBA)$_{18}$ and $Au_{25}/BaLa_4Ti_4O_{15}$ obtained by calcination at 250 °C and 300 °C together with that of Au foil.

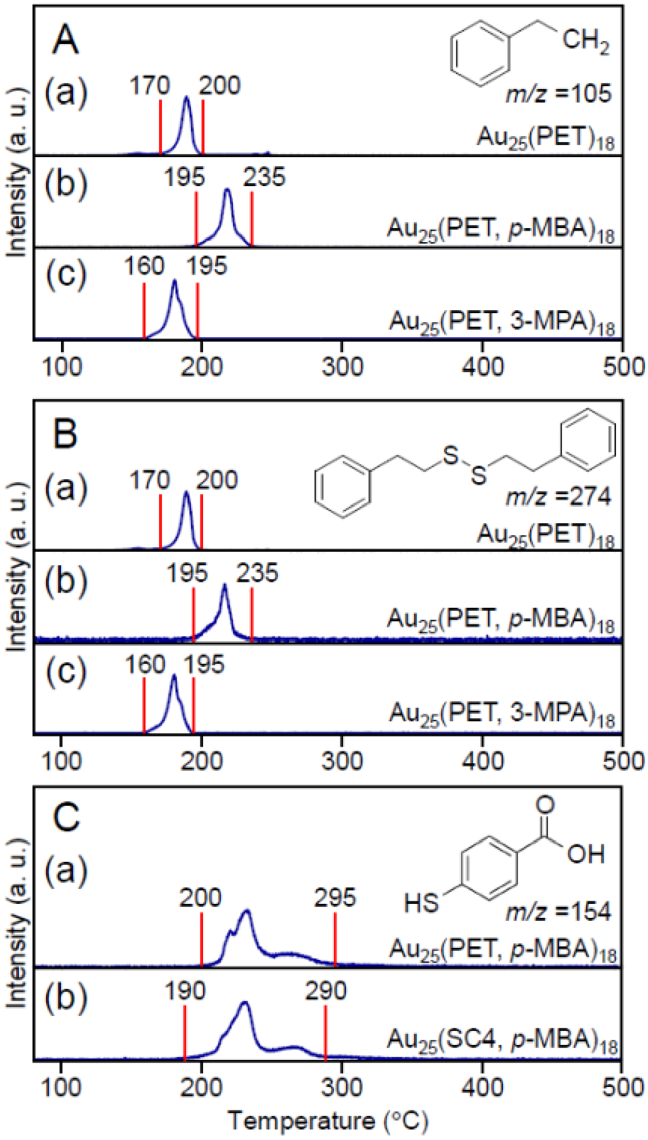

Figure S12. Temperature dependence of each mass peak: (A) PE, (B) $(PET)_2$, and (C) *p*-MBA depending on the starting Au_{25} NCs. In (A) and (B), the starting Au_{25} NCs is (a) $Au_{25}(PET)_{18}$, (b) $Au_{25}(PET, p\text{-MBA})_{18}$, and (c) $Au_{25}(PET, 3\text{-MPA})_{18}$. In (C), the starting Au_{25} NCs is (a) $Au_{25}(PET, p\text{-MBA})_{18}$ and (b) $Au_{25}(SC4, p\text{-MBA})_{18}$. These results indicate that the temperature required for desorption depends on the combination of ligands, demonstrating that in addition to the binding energy of each bond, the magnitude of the ligand interaction on the Au_{25} surface also significantly affects the desorption temperature of the ligands.

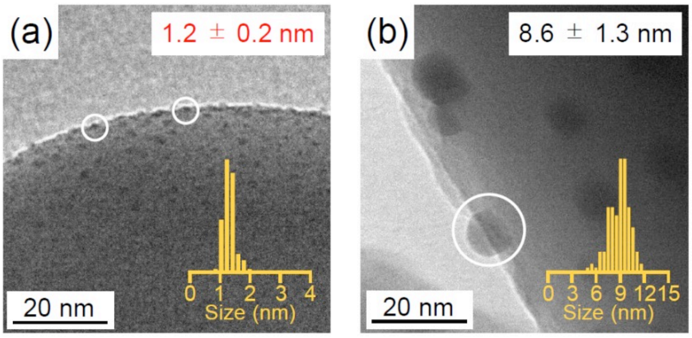

Figure S13. TEM images of the samples obtained by the calcination of $Au_{25}(PET, p\text{-MBA})_{18}/BaLa_4Ti_4O_{15}$ at 300 °C under (a) reduced pressure (Figure 6(e)) and (b) oxygen atmosphere. Typically, the ligands are burned quickly, namely at low temperature, in the calcination under oxygen atmosphere, which seems to lead to the remarkably aggregation of Au_{25} in the sample of (b). This result demonstrates that the degree of aggregation of Au_{25} at a particular temperature varies depending on the calcination atmosphere.

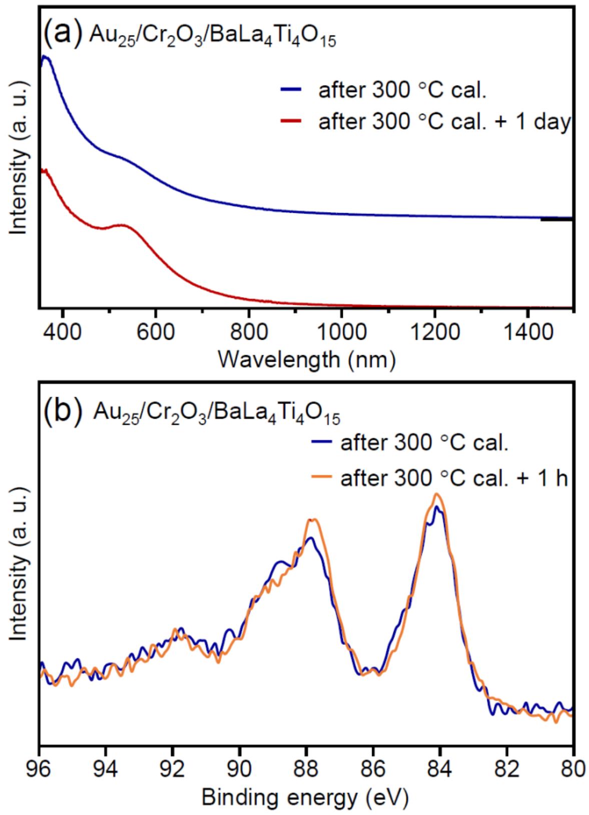

Figure S14. Time-dependence of (a) DR spectrum and (b) Au 4f XPS spectrum for $Au_{25}/Cr_2O_3/BaLa_4Ti_4O_{15}$ obtained by the calcination at 300 °C. In (a), the absorption at 520 nm, which is assignable to plasmon peak,[8] increased after one day in air. The plasmon peak is typically observed for the Au NCs with the size of >2 nm, indicating that the $Au_{25}/Cr_2O_3/BaLa_4Ti_4O_{15}$ after 1 day includes the Au NCs with the size of >2 nm. In (b), the peak structure slightly approached to that of Au foil after 1 h in vacuum. This can be seen through the decrease in intensity at the high binding energy side of the Au $4f_{7/2}$ and $4f_{5/2}$ peak. These results indicate that the bare Au_{25} is unstable on metal oxide and therefore easily aggregate both in air and vacuum.

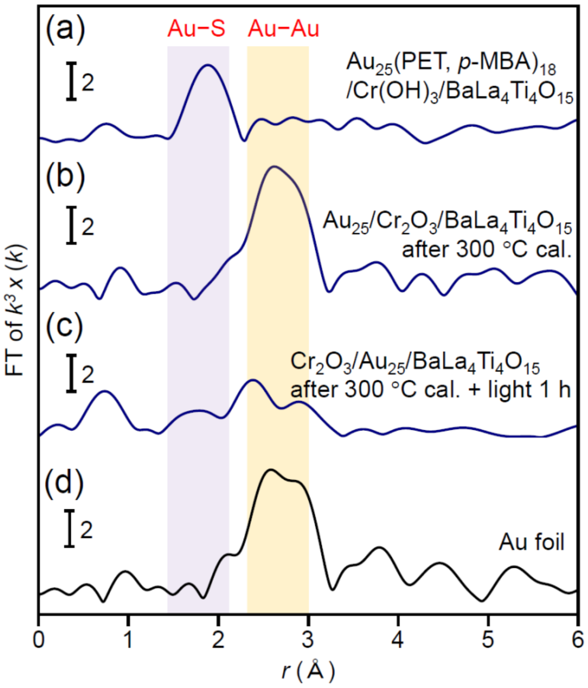

Figure S15. Au L_3-edge FT-EXAFS spectra of (a) Au_{25}(PET, *p*-MBA)$_{18}$/Cr(OH)$_3$/$BaLa_4Ti_4O_{15}$ and (b) $Au_{25}/Cr_2O_3/BaLa_4Ti_4O_{15}$ obtained by calcination at 300 °C, (c) $Cr_2O_3/Au_{25}/BaLa_4Ti_4O_{15}$ obtained by UV-light irradiation, and (d) Au foil. In the figure, purple and yellow regions indicate the Au−S and Au−Au bond region, respectively. In (b)(c), the Au−S bond was not observed, indicating that most of Au−S bonds were dissociated by the calcination at 300 °C, similar to the case of Au_{25}(PET, *p*-MBA)$_{18}$/$BaLa_4Ti_4O_{15}$. In (b), the Au−Au bond was clearly observed because of the aggregation of Au_{25} in this sample (see Figure 9A(a)). On the other hand, in (c), the Au−Au bond was observed with lower intensity because of lesser aggregation of Au_{25} in this sample compared to the sample in (b) (see Figure 9A(b)).

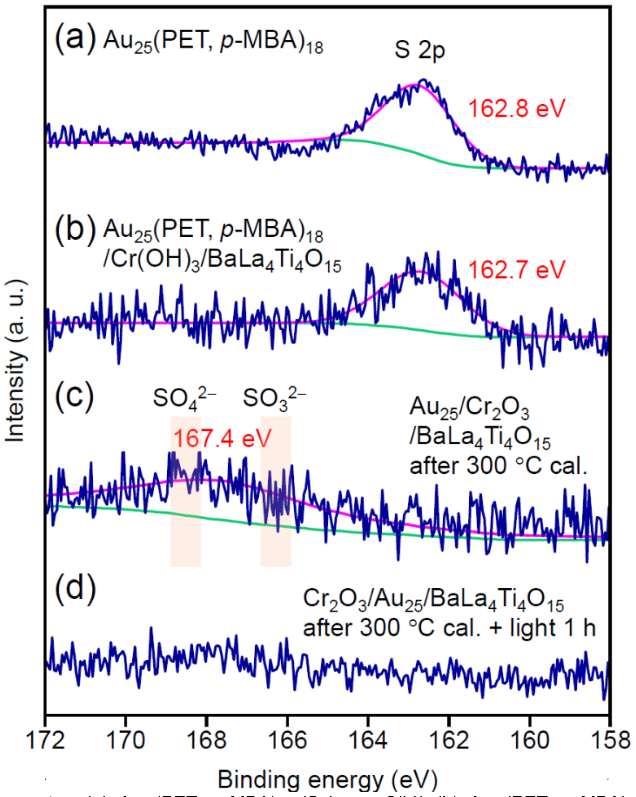

Figure S16. Comparison of S 2p XPS spectra; (a) Au_{25}(PET, *p*-MBA)$_{18}$ (Scheme 2(b)) (b) Au_{25}(PET, *p*-MBA)$_{18}$/$Cr(OH)_3$/$BaLa_4Ti_4O_{15}$ (Scheme 2(c)), (c) Au_{25}/Cr_2O_3/$BaLa_4Ti_4O_{15}$ obtained by calcination at 300 °C (Scheme 2(d)), and (d) Cr_2O_3/Au_{25}/$BaLa_4Ti_4O_{15}$ obtained by UV-light irradiation (Scheme 2(e)). In the spectra, green and purple lines indicate the baseline and fitting result, respectively. Figure (c) demonstrates that the Au_{25}/Cr_2O_3/$BaLa_4Ti_4O_{15}$ contained S in the form of SO_3^{2-} or SO_4^{2-}.[9] Figure (d) demonstrates that most of SO_3^{2-} or SO_4^{2-} were eliminated from Cr_2O_3/Au_{25}/$BaLa_4Ti_4O_{15}$. It can be considered that most of SO_3^{2-} or SO_4^{2-} were reduced by photogenerated charge and then the resulting H_2S is washed away with water from Cr_2O_3/Au_{25}/$BaLa_4Ti_4O_{15}$. However, based on the EDX elemental mapping obtained by HAADF-STEM image (Figure 9B and S22 and Table S2), a small quantity of sulfur remains in the sample even after UV-light irradiation.

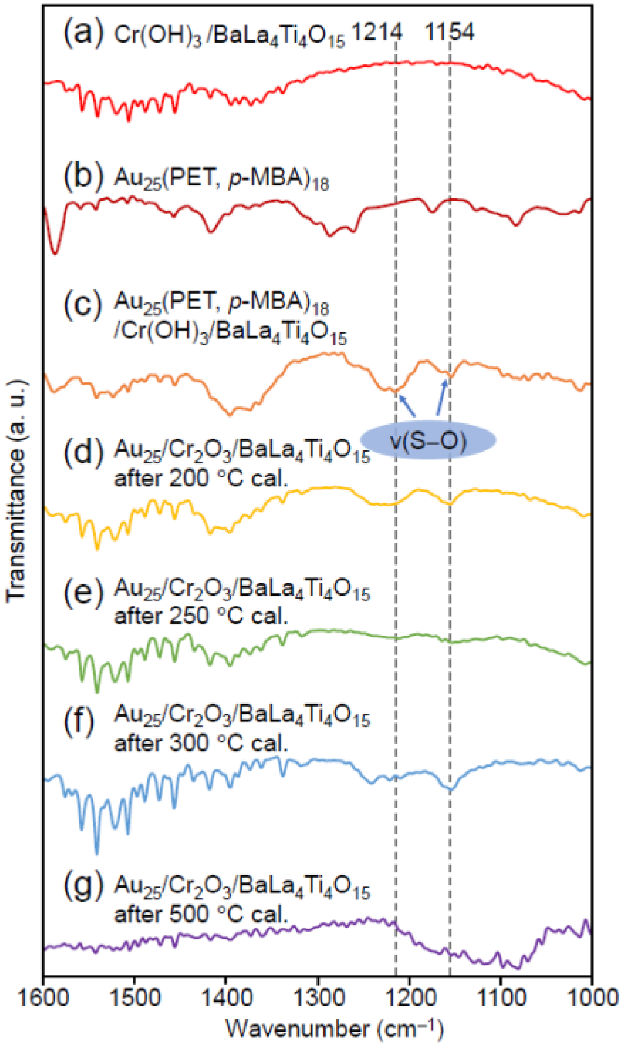

Figure S17. Comparison of FT-IR spectra: (a) $Cr(OH)_3/BaLa_4Ti_4O_{15}$, (b) Au_{25}(PET, *p*-MBA)$_{18}$, (c) Au_{25}(PET, *p*-MBA)$_{18}$/$Cr(OH)_3/BaLa_4Ti_4O_{15}$, and $Au_{25}/Cr_2O_3/BaLa_4Ti_4O_{15}$ obtained by the calcination at (d) 200 °C, (e) 250 °C, (f) 300 °C, and (e) 500 °C. In these measurements, 0.5 wt% Au was loaded on $Cr(OH)_3/BaLa_4Ti_4O_{15}$ to easily monitor the S−O stretching peaks (1154 and 1214 cm^{-1}).[10] The peaks attributed to S−O stretching (1154 and 1214 cm^{-1}) do not appear in the spectra of $Cr(OH)_3/BaLa_4Ti_4O_{15}$ (a) and unsupported Au_{25}(PET, *p*-MBA)$_{18}$ (b). On the other hand, in the spectrum of the sample before calcination (c), a peak attributed to S−O stretching was observed. These results indicate that some of the ligands in Au_{25}(PET, *p*-MBA)$_{18}$/$Cr(OH)_3/BaLa_4Ti_4O_{15}$ migrated from Au_{25}(PET, *p*-MBA)$_{18}$ to $Cr(OH)_3/BaLa_4Ti_4O_{15}$ without heating. Such ligand migration is interpreted to be related to the start of the styrene desorption at 150 °C in Au_{25}(PET, *p*-MBA)$_{18}$/$Cr(OH)_3/BaLa_4Ti_4O_{15}$. In (f), the peaks assignable to S−O stretching peaks were not observed, implying that almost all the S compounds were eliminated from the $Cr_2O_3/BaLa_4Ti_4O_{15}$ surface by the calcination at 500 °C.

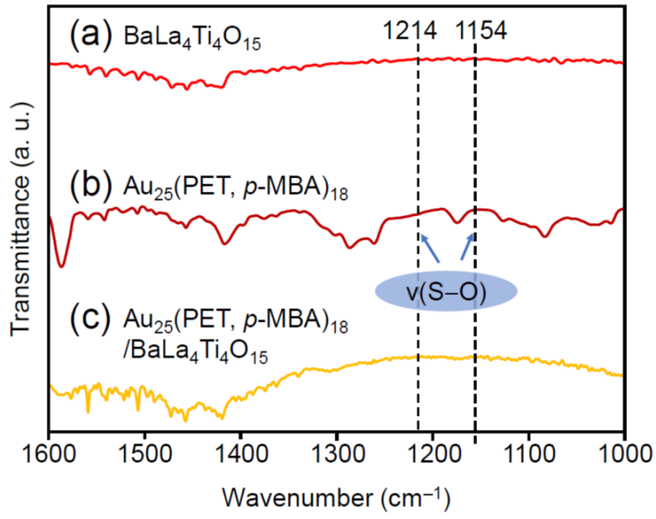

Figure S18. Comparison of FT-IR spectra; (a) $BaLa_4Ti_4O_{15}$, (b) Au_{25}(PET, *p*-MBA)$_{18}$, and (c) Au_{25}(PET, *p*-MBA)$_{18}$/$BaLa_4Ti_4O_{15}$. In these measurements, 0.5 wt% Au was loaded on $BaLa_4Ti_4O_{15}$ to easily monitor the S−O stretching peaks (1154 and 1214 cm^{-1})[10]. In Figure (c), the peaks assignable to S−O stretching peaks are not observed, implying that the migration of $(PET)_2$, *p*-MBA, and/or PET−*p*-MBA from Au_{25}(PET, *p*-MBA)$_{18}$ to $BaLa_4Ti_4O_{15}$ did not occur in Au_{25}(PET, *p*-MBA)$_{18}$/$BaLa_4Ti_4O_{15}$, different from the case of Au_{25}(PET, *p*-MBA)$_{18}$/$Cr(OH)_3$/$BaLa_4Ti_4O_{15}$ (Figure S17). These results indicate that Cr-oxide layer on $BaLa_4Ti_4O_{15}$ induces the migration of $(PET)_2$, *p*-MBA, and/or PET−*p*-MBA from Au_{25}(PET, *p*-MBA)$_{18}$ to the metal oxide. It is considered that the early start of the desorption of styrene (150 °C) in Au_{25}(PET, *p*-MBA)$_{18}$/$Cr(OH)_3$/$BaLa_4Ti_4O_{15}$ (Figure 8B) is caused by this easiness of the migration of $(PET)_2$, *p*-MBA, and/or PET−*p*-MBA from Au_{25}(PET, *p*-MBA)$_{18}$ to the metal oxide.

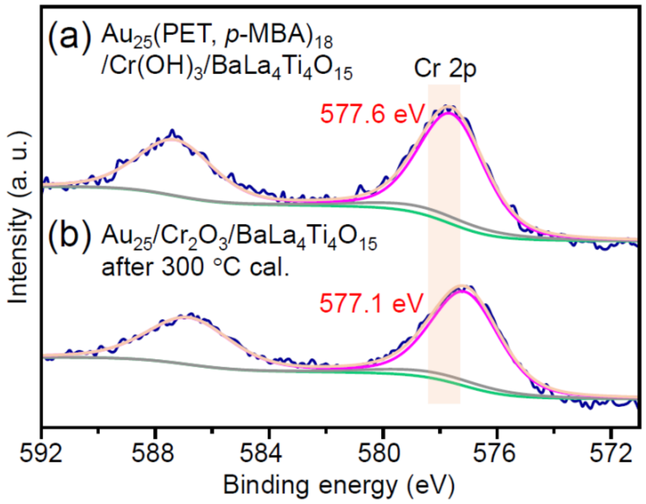

Figure S19. Comparison of Cr 2p XPS spectra of (a) Au_{25}(PET, *p*-MBA)$_{18}$/$Cr(OH)_3$/$BaLa_4Ti_4O_{15}$ and (b) Au_{25}/Cr_2O_3/$BaLa_4Ti_4O_{15}$ obtained by calcination at 300 °C. In the spectra, green and purple lines indicate the baseline and fitting result, respectively. Before the calcination (Au_{25}(PET, *p*-MBA)$_{18}$/$Cr(OH)_3$/$BaLa_4Ti_4O_{15}$), the peak appeared at 577.6 eV, implying that Cr-oxide layer is mainly composed of $Cr(OH)_3$.[9] After calcination (Au_{25}/Cr_2O_3/$BaLa_4Ti_4O_{15}$), the peak is found at 577.1 eV, implying that Cr-oxide layer is mainly composed of Cr_2O_3.[9] Based on this result, we changed our interpretation on the chemical composition of a Cr-oxide layer from the previous one[2,4]: In the previous papers,[2,4] we have described that Cr_2O_3 layer was loaded on $BaLa_4Ti_4O_{15}$ by photodeposition method. There should be a difference in the compound−support interaction between Au_{25}(PET, *p*-MBA)$_{18}$/$Cr(OH)_3$/$BaLa_4Ti_4O_{15}$ and Au_{25}(PET, *p*-MBA)$_{18}$/$BaLa_4Ti_4O_{15}$. This difference might be related to the difference in the compound desorption temperatures of the two catalysts (Figure 3B vs. Figure 8B).

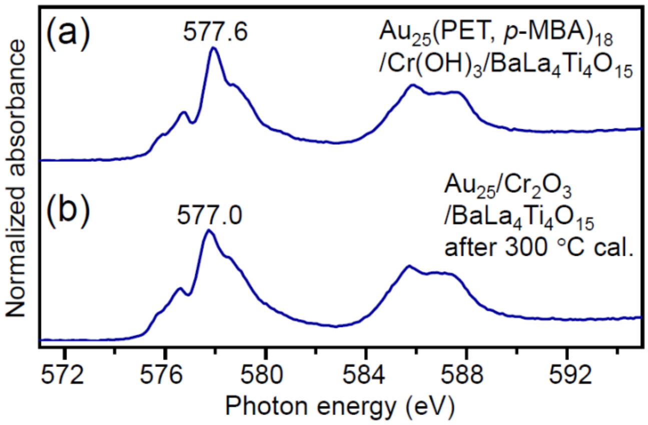

Figure S20. Cr $L_{3,2}$-edge XANES spectra of (a) Au_{25}(PET, *p*-MBA)$_{18}$/$Cr(OH)_3$/$BaLa_4Ti_4O_{15}$ and (b) Au_{25}/Cr_2O_3/$BaLa_4Ti_4O_{15}$ obtained by calcination at 300 °C. Before the calcination (Au_{25}(PET, *p*-MBA)$_{18}$/$Cr(OH)_3$/$BaLa_4Ti_4O_{15}$), the peak appeared at 577.6 eV, implying that chromium-oxide layer mainly composed of $Cr(OH)_3$.[11] After calcination (Au_{25}/Cr_2O_3/$BaLa_4Ti_4O_{15}$), the peak is found at 577.0 eV, implying that chromium-oxide layer mainly composed of Cr_2O_3. Based on this result, we changed our interpretation on the chemical composition of a Cr-oxide layer from the previous one[2,4]: In the previous papers,[2,4] we have described that Cr_2O_3 layer was loaded on $BaLa_4Ti_4O_{15}$ by photodeposition method. There should be a difference in the compound−support interaction between Au_{25}(PET, *p*-MBA)$_{18}$/$Cr(OH)_3$/$BaLa_4Ti_4O_{15}$ and Au_{25}(PET, *p*-MBA)$_{18}$/$BaLa_4Ti_4O_{15}$. This difference might be related to the difference in the compound desorption temperatures of the two catalysts (Figure 3B vs. Figure 8B).

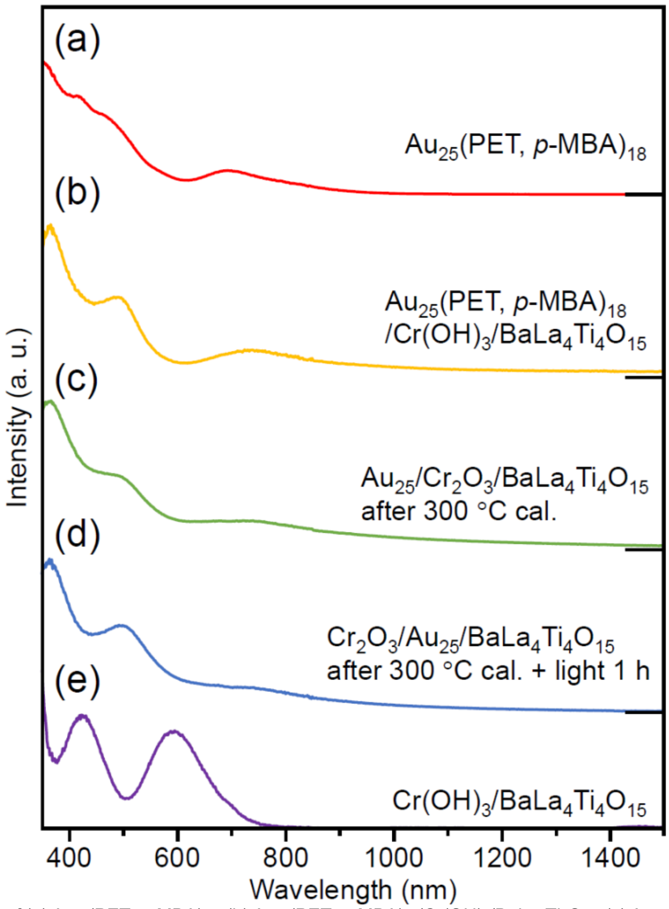

Figure S21. Diffuse reflectance spectra of (a) Au_{25}(PET, *p*-MBA)$_{18}$, (b) Au_{25}(PET, *p*-MBA)$_{18}$/$Cr(OH)_3$/$BaLa_4Ti_4O_{15}$, (c) Au_{25}/Cr_2O_3/$BaLa_4Ti_4O_{15}$ obtained by calcination at 300 °C (d) Cr_2O_3/Au_{25}/$BaLa_4Ti_4O_{15}$ by UV-light irradiation, and (e) $Cr(OH)_3$/$BaLa_4Ti_4O_{15}$. In (b−d), the absorption due to $Cr(OH)_3$/$BaLa_4Ti_4O_{15}$ is subtracted. These results demonstrate that the electronic structure of Au NCs is changed by calcination (ligand elimination).

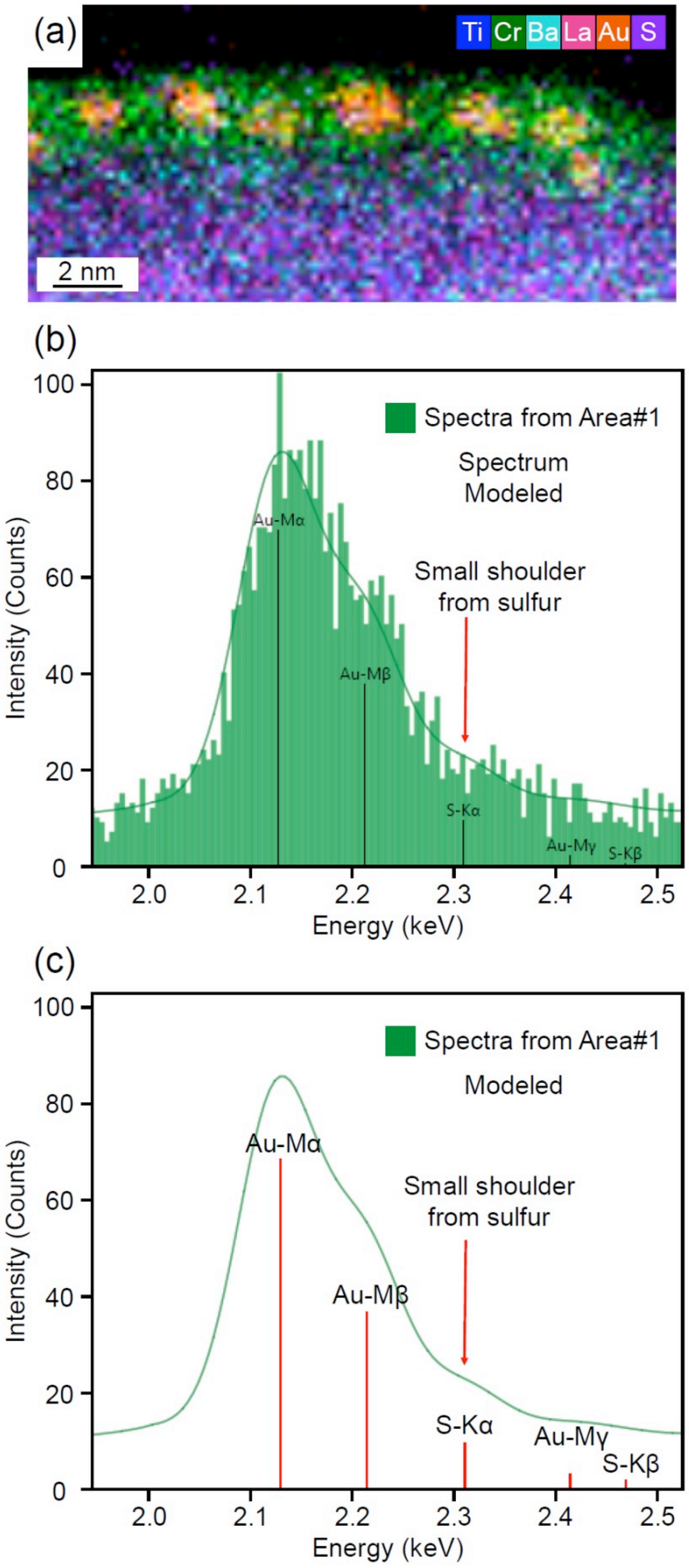

Figure S22. Result of EDX elemental-analysis for the sample of Figure 9B (Table S2); (a) Area used for the analyses and (b,c) the results of analyses. Because the peak position of sulfur is near to that of Au, a quantitative determination of sulfur is not easy, however, it is clear that small amounts of sulfur do remain in the samples.

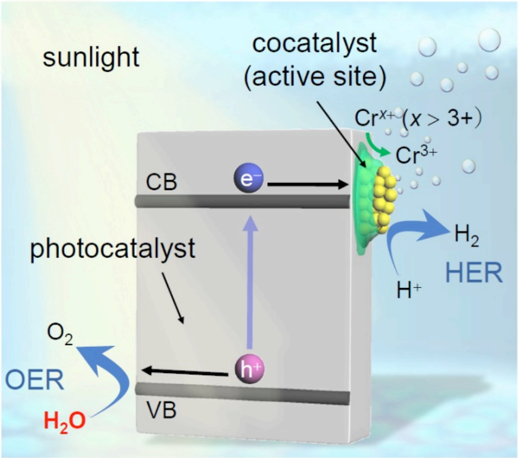

Figure S23. Proposed mechanism of the formation of the chromium-oxide shell on Au NCs. The electrons in the conduction band (CB), which were photoinduced by UV-light irradiation, is considered to be used for the reduction of highly oxidized Cr ions (> 3+)[4] as well as the evolution of hydrogen (H_2) (H_2-evolution reaction; HER). It seems that highly oxidized Cr ions (> 3+) moves to the top of the Au NCs during this reaction since the reduction occurs on the surface of bare Au NCs, leading to the formation of Cr-oxide shells on Au NCs. Based on this mechanism, it is expected that the reaction speed of the shell formation can be accelerated by adding the sacrificial reagent, which effectively consumes the photoinduced hole.

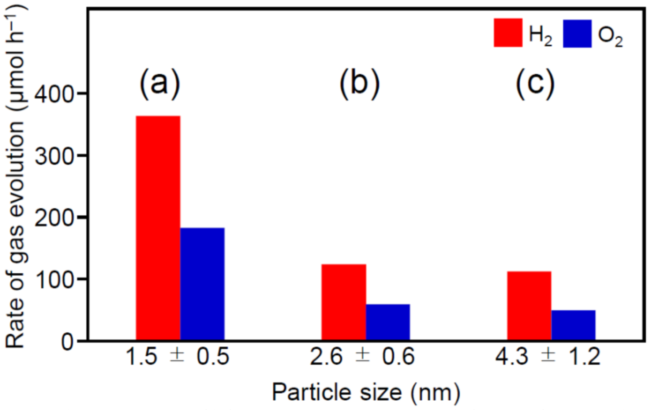

Figure S24. Rates of photocatalytic generation of H_2 and oxygen (O_2) by water splitting with Cr_2O_3/Au NCs/$BaLa_4Ti_4O_{15}$ having Au NC particle-size of (a) 1.5 ± 0.5 (Figure 9A(b)), (b) 2.6 ± 0.6, and (c) 4.3 ± 1.2 nm. Sample (a)−(c) were prepared by the calcination at 300 °C under the reduced pressure, air, and O_2 atmosphere. All the samples include 0.1 wt% Au and were treated with UV-light irradiation. This result indicates that the water-splitting activity increases with decrease of Au particle-size.

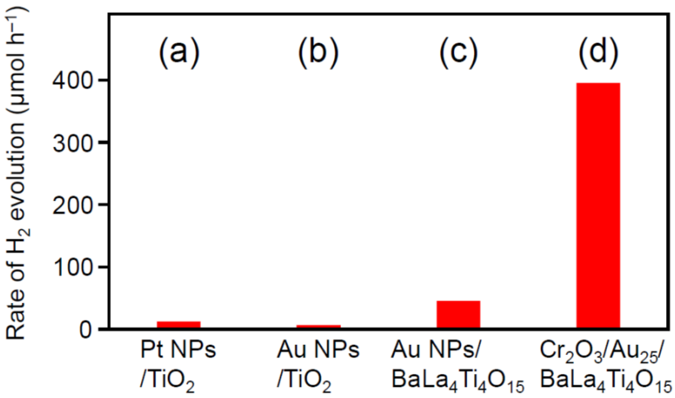

Figure S25. Rates of photocatalytic generation of H_2 by water splitting with (a) Pt NPs/TiO_2 (12.41 μmol h^{-1}), (b) Au NPs/TiO_2 (6.48 μmol h^{-1}), (c) Au NPs/$BaLa_4Ti_4O_{15}$ (45.14 μmol h^{-1}), and (d) Cr_2O_3/Au_{25}/$BaLa_4Ti_4O_{15}$ (395.11 μmol h^{-1}; this work). Pt NPs/TiO_2, Au NPs/TiO_2, Au NPs/$BaLa_4Ti_4O_{15}$ were prepared by the photodeposition method with loading weight of 1.0 wt% metal.[12] This result indicates that the photocatalysts created in this study exhibit higher performance than previously used photocatalyst.

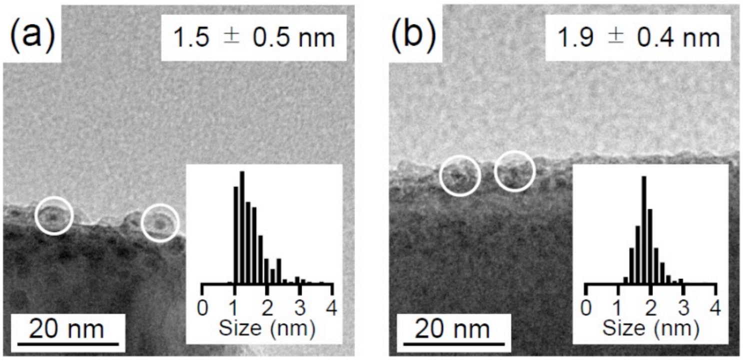

Figure S26. TEM images of Cr_2O_3/Au NCs/$BaLa_4Ti_4O_{15}$ with Au NC particle-size of 1.5 ± 0.5 nm (Figure 9A(b)) (a) before and (b) after the water-splitting reaction for 10 h.

References

[1] H.Qian, C. Liu, R. Jin, *Sci. China: Chem*. **2012**, *55*, 2359–2365.

[2] W. Kurashige, R. Hayashi, K. Wakamatsu, Y. Kataoka, S. Hossain, A. Iwase, A. Kudo, S. Yamazoe, Y. Negishi, *ACS Appl. Energy Mater.* **2019**, *2*, 4175–4187.

[3] Y. Miseki, H. Kato, A. Kudo, *Energy Environ. Sci.* **2009**, *2*, 306–314.

[4] W. Kurashige, R. Kumazawa, D. Ishii, R. Hayashi, Y. Niihori, S. Hossain, L. V. Nair, T. Takayama, A. Iwase, S. Yamazoe, T. Tsukuda, A. Kudo, Y. Negishi, *J. Phys. Chem. C* **2018**, *122*, 13669–13681.

[5] Y. Negishi, M. Mizuno, M. Hirayama, M. Omatoi, T. Takayama, A. Iwase, A. Kudo, *Nanoscale* **2013**, *5*, 7188–7192.

[6] H. Asakura, S. Yamazoe, T. Misumi, A. Fujita, T. Tsukuda, T. Tanaka, *Radiat. Phys. Chem.* **2020**, *175*, 108270.

[7] D. P. Anderson, J. F. Alvino, A. Gentleman, H. A. Qahtani, L. Thomsen, M. I. J. Polson, G. F. Metha, V. B. Golovko, G. G. Andersson, *Phys. Chem. Chem. Phys.* **2013**, *15*, 3917–3929.

[8] Y. Negishi, T. Nakazaki, S. Malola, S. Takano, Y. Niihori, W. Kurashige, S. Yamazoe, T. Tsukuda, H. Häkkinen, *J. Am. Chem. Soc.* **2015**, *137*, 1206–1212.

[9] C. D. Wagner, W. M. Riggs, L. E. Davis, J. F. Moulder, 1st ed.; Physical Electronics Division, Perkin-Elmer Corp.: Eden Prairie, MN, **1979**.

[10] H. A. Almukhlifi, R. A. Burns, *J. Mol. Catal. A: Chem.* **2016**, *411*, 349–363.

[11] A. S. Alotabi, C. T. Gibson, G. F. Metha, G. G. Andersson, *ACS Appl. Energy Mater* **2021**, *4*, 322–330.

[12] Y. Lin, Y. Liu, Y. Li, Y. Cao, J. Huang, H. Wang, H. Yu, H. Liang, F. Peng, *ACS Sustainable Chem. Eng.* **2018**, *6*, 17340–17351.
